# Supplementary material for: Genome-wide meta-analyses of stratified depression in Generation Scotland and UK Biobank
Source: Transl Psychiatry. 2018 Jan 10;8:9. doi: 10.1038/s41398-017-0034-1 (PMC5802463; doi:10.1038/s41398-017-0034-1)
Supplement: Supplementary file 1 — Supplementary materials [file 41398_2017_34_MOESM1_ESM.docx]

**Supplementary materials**

**This document contains supplementary material for Hall et al - Genome-wide meta-analyses of stratified depression in Generation Scotland and UK Biobank**

**Supplementary Figures and Tables**

**Supplementary Figure 1. Scree plots for up to 20 ancestral components in depression subsets 10**

**Supplementary Figure 2. Age distributions for parents and mid-parents from two and three generation families 11**

**Supplementary Figure 3. QQ plot for meta-analysis of MDD, rMDD, fMDD and mMDD 14**

**Supplementary Figure 4. Regional association plot for rs4478037 in MDD subsets 23**

**Supplementary Figure 5. Conditional meta-analysis for rs4478037 in mMDD 24**

**Supplementary Table 1. Demographic information in GS:SFHS, UKB, and population-based estimates for depression subsets 6**

**Supplementary Table 2. Significant ancestral components for depression subsets 10**

**Supplementary Table 3. Testing shared environmental effects 13**

**Supplementary Table 4. Meta-analysis summary statistics from GWAS of depression 15**

**Supplementary Table 5. Association results from GWAS of depression in datasets separately 16**

**Supplementary Table 6. Meta-analysis summary statistics from recurrent cases only GWAS 17**

**Supplementary Table 7. Association results from recurrent cases only GWAS in datasets 18 separately**

**Supplementary Table 8. Meta-analysis summary statistics from females only GWAS 19**

**Supplementary Table 9. Association results from females only GWAS in datasets separately 20**

**Supplementary Table 10. Meta-analysis summary statistics from males only GWAS 21**

**Supplementary Table 11. Association results from males only GWAS in datasets separately 22**

**Supplementary Table 12. MAGMA gene-based 25**

**Supplementary Table 13. DEPICT reconstituted gene set enrichment 25**

**Supplementary Table 14. Functional annotation of lead SNPs using GTEx and RegulomeDB databases 26-27**

**Supplementary Table 15. SNP-based heritability using GCTA-GREML 28**

**Supplementary Table 16. Polygenicity and SNP-based heritability using LD Score Regression 29**

**Supplementary Table 17. Genetic correlation between traits using LD Score Regression 30-32**

**Supplementary Table 18. Polygenic profiling analysis of MDD in GS:SFHS using PGCMDD weights 33**

**Supplementary Table 19. Polygenic profiling analysis of MDD in GS:SFHS using UKB weights 34**

**Supplementary Table 20. Polygenic profiling analysis of MDD in UKB using PGCMDD weights 35**

**Supplementary Table 21. Polygenic profiling analysis of MDD in UKB using GS:SFHS weights 36**

[**Supplementary methods** 3](#_Toc479628544)

[Sample collection, genotyping and assessment of depression in Generation Scotland: The Scottish Family Health Study (GS:SFHS) and UK Biobank (UKB) 3](#_Toc479628545)

[Genome-wide association analysis in GS:SFHS 6](#_Toc479628546)

[Pathway and functional genomic analyses 7](#_Toc479628547)

[Polygenic profiling analysis 9](#_Toc479628548)

[Accounting for population stratification using principal components 9](#_Toc479628549)

[Accounting for shared environmental factors in GS:SFHS 11](#_Toc479628550)

[**Supplementary Results** 14](#_Toc479628551)

[Genome wide association analysis 14](#_Toc479628552)

[Gene-based analysis 25](#_Toc479628553)

[Pathway and functional genomic analysis 25](#_Toc479628554)

[Estimating SNP-based heritability using GREML 28](#_Toc479628555)

[Estimating polygenicity and SNP-based heritability using Linkage Disequilibrium Score Regression (LDSR) 29](#_Toc479628556)

[Genetic correlation with health related traits 30](#_Toc479628557)

[Polygenic profiling analysis 33](#_Toc479628558)

[Major Depressive Disorder Working Group of the Psychiatric Genomics Consortium Authorship 37](#_Toc479628559)

[References 44](#_Toc479628560)

# Supplementary methods

# Sample collection, genotyping and assessment of depression in Generation Scotland: The Scottish Family Health Study (GS:SFHS) and UK Biobank (UKB)

## Sample collection in GS:SFHS

GS:SFHS is a family and population-based study consisting of 23,960 participants, aged between 18 and 99, recruited via general medical practices across Scotland between 2006 and 2011. Potential participants were identified from the registers of collaborating general practices via their Community Health Index, a unique identifying number allocated to nearly every individual in Scotland registered with a general practitioner. A shared family identity number was given to groups where each member was a first-degree relative of at least one other person, resulting in 5,573 families. Family size ranged from 2 - 36 members, with a mean family size (excluding 1,400 singletons without any relations in the study) of 4.05 members and a median family size of 3 (IQR 2–5)(1, 2).

## Genotyping in GS:SFHS

Blood samples were obtained using standard operating procedures and were stored at the Wellcome Trust Clinical Research Facility Genetics Core (www.wtcrf.ed.ac.uk). DNA extraction was performed following methods described elsewhere(3). Genotyping was performed using the Illumina HumanOmniExpressExome-8 v1.0 DNA Analysis BeadChip and Infinium chemistry. Genotypes were processed using GenomeStudio Analysis Software v2011.1(4).

## Measurement and definition of depression in GS:SFHS

A diagnosis of depression was made using the structured clinical interview for DSM-IV disorders (SCID)(5). Researchers administered the screening questions (“Have you ever seen anybody for emotional or psychiatric problems?” and “Was there ever a time when you, or someone else, thought you should see someone because of the way you were feeling or acting?”) after a period of training and reliability assessment. Participants who answered yes to either of these questions (21.7% screened positive) were invited to continue the interview, which provided information on the presence or absence of a lifetime history of depression, age of onset and number of depressive episodes. Inter-rater reliability for the presence or absence of a lifetime diagnosis of major depressive disorder was high (κ = 0.86, p < 0.001, 95%CI = 0.7-1.0)(6).

## Sample collection in UKB

UKB is a population-based study consisting of over 500,000 participants aged 40-69 recruited via invitation across the United Kingdom between 2006 and 2010. Potential participants were identified from NHS patient registers and as living within a reasonable travelling distance of an assessment centre(7, 8).

## Genotyping in UKB

The first genotype data release of UK Biobank samples (n=152,729) were genotyped using either the Affymetrix UK BiLEVE Axiom array (n=49,979) or the Affymetrix UK Biobank Axiom array (n=102,750). These arrays have over 95% content in common. Genotyping was performed on 33 batches of ~4700 samples by Affymetrix (High Wycombe, UK). Only autosomal data were available under the current data release. Further details of the sampling process in UKB are available at http://biobank.ctsu.ox.ac.uk/crystal/refer.cgi?id=155583, and the Axiom array at http://media.affymetrix.com/support/downloads/manuals/axiom_2_assay_auto_workflow_user_guide.pdf.

## Measurement and definition of depression in UKB

Questions on depressive and manic symptoms were administered via a touchscreen questionnaire(9). These questions were only added to the assessment protocol in the last two years of recruitment, resulting in information from 172,751 participants - 149,847 of whom had sufficient data to allow an assessment of probable major depressive disorder and/or probable bipolar disorder. Current and previous depression symptoms were assessed by items relating to the lifetime experience of minor and major depression, items from the Patient Health Questionnaire(10) and items on help-seeking for mental health. A probable history of bipolar disorder was determined using questions assessing manic symptoms from the SCID(5). Criteria for depression were based largely on questions assessing low mood and anhedonia as core symptoms, and having been assessed by a General Practitioner or psychiatrist ("Looking back over your life, have you ever had a time when you were feeling depressed or down for at least a whole week?", "Have you ever seen a psychiatrist for nerves, anxiety, tension or depression?", "Have you ever had a time when you were uninterested in things or unable to enjoy the things you used to for at least a whole week?"). Individuals were excluded if they had mild depressive or manic symptoms, which entailed that individuals were not assessed by a health professional for any criteria symptoms they had experienced(11). Further exclusions were made on the basis of touchscreen data and health records. Participants were excluded as cases and controls if they had ever had a diagnosis from health records of bipolar disorder, multiple personality disorder, schizophrenia, autism, intellectual disability, or Parkinson’s disease; if they had ever had a prescription for antipsychotic or mood stabilising medication; if they self-reported bipolar disorder, schizophrenia, or Parkinson’s disease; or if a touchscreen questionnaire assessment indicated bipolar disorder(11). Additionally, participants were excluded as controls if they had ever had a diagnosis of an anxiety disorder, a mood disorder, or major depressive disorder; if they had ever been prescribed antidepressant or anxiolytic medication; or if they self-reported depression.

## Demographic distributions in GS:SFHS and UKB

In terms of disease prevalence, GS:SFHS is consistent with the epidemiological literature prevalence estimates for depression (MDD)(12), recurrent depression (rMDD)(13), depression in females only (fMDD)(14) and depression in males only (mMDD)(14). The prevalence of MDD (and consequently rMDD, fMDD and mMDD) in UKB are much higher than both GS:SFHS and the UK population(15). This is due to the exclusion of large numbers of controls, owing to incomplete and missing data, and a lower threshold for case definition(11), relative to GS:SFHS(1, 2). The proportion of males in UKB is consistent with the population average(15), however GS:SFHS has a considerably lower proportion of males. Both GS:SFHS and, particularly, UKB have a higher median age relative to the general population(15) This is due to both cohorts being primarily (GS:SFHS) or exclusively (UKB) recruited at middle age(1, 2, 11). In both GS:SFHS and UKB, case group had a significantly higher proportion of females (71.4% and 62.3%, respectively) than the control group (56.8% and 43.6%, respectively), as determined by the Pearson's χ^2^ test (GS:SFHS
χ^2^ = 198.6, df = 1, p-value <2.2e-16; UKB χ^2^ = 761.82, df = 1, p-value <2.2e-16). This is consistent with the epidemiological literature, where the lifetime prevalence is estimated as being approximately two times greater in females than males(14). Demographic distributions for GS:SFHS, UKB and the UK general population are shown in Supplementary Table 1.

| **Cohort** | **MDD prev.  (N)** | **rMDD prev. (N)** | **fMDD prev. (N)** | **mMDD prev. (N)** | **Prop. males** | **Median age (IQR)** |
| --- | --- | --- | --- | --- | --- | --- |
| GS | 0.139 (2 603; 16 122) | 0.074  (1 289; 16 122) | 0.169  (1 859;9 159) | 0.096  (742; 6 958) | 0.41 | 49.0  (36.0-59.0) |
| UKB | 0.339 (8 248; 16 089) | 0.273 (6 056; 16 089) | 0.423 (5 138;7 013) | 0.255 (3 110;9 076) | 0.50 | 60.0  (40.0-64.0) |
| Total | 0.252  (10 851; 32 211) | 0.186  (7 345; 32 211) | 0.302  (6 997;16 172) | 0.194  (3 852;16 034) | 0.46 | 56.0 (46.0-63.0) |
| Pop. | 0.162(12) | 0.069(13) | 0.174(14) | 0.070(14) | 0.49(15) | 40.0  (21.1-58.3)(15) |

Supplementary Table 1. Demographic information on disease prevalence for MDD (all cases and controls, MDD; recurrent cases and all controls, rMDD; female cases and controls, fMDD and male cases and controls, mMDD) subtypes in Generation Scotland (GS:SFHS), UK Biobank (UKB) and populations of European descent, with the number of cases and controls (respectively) in parentheses; sex distributions and age distributions in GS:SFHS, UKB and the UK population average (sex and age). Sex-specific prevalence estimates were derived by averaging across estimates from French, German and Italian populations(14).

# Genome-wide association analysis in GS:SFHS

Genome-wide association analysis (GWAS) of MDD, rMDD, fMDD and mMDD in GS:SFHS were conducted using mixed linear model based association (MLMA) analysis(16), implemented in GCTA (v1.25.)(17). The mixed linear model used can be written as:

**y**= **Xβ** + **Zu_1_** + **Zu_2_** + **ε**

where y is the vector of binary observations for MDD, rMDD, fMDD or mMDD. β is a matrix of fixed effects, here the additively coded allele. u_1_ and u_2_ are random effects taking into account the genomic relationships, following a multivariate normal distribution of mean 0, and variance
Gσ, where G is the genomic relationship matrix. X, Z, and Z are design matrices of regressors which relate the observations of y to β, u_1_ and u_2_ respectively. ε is an unknown vector of random errors, and assumed to follow a multivariate normal distribution of mean zero and variance Iσ, where I is an identity matrix. Two genomic relationship matrices (GRMs) were used to account for population structure, as this method has been demonstrated by Zaitlen *et al* to account for potential upward biases due to excessive relationships, thus allowing the inclusion of closely and distantly related individuals in genetic analyses(18). The first GRM included pairwise relationship coefficients for all individuals. The second GRM had off-diagonal elements of pairs of individuals who had a relationship coefficient < 0.05 set to 0, therefore excluding pairs of individuals that have a most recent common ancestor of approximately four generations distant, assuming no inbreeding(19). GRMs were created using the mixed linear model with candidate marker excluded (MLMe) approach(16). The MLMe approach involves removing SNPs from the chromosome in question, from the base set of 536,560 genotyped autosomal SNPs, therefore creating an MLMe GRM for each chromosome. This was to prevent loss of power through double fitting of the candidate SNP (and those in LD with it) in the GRM as a random effect, while testing each SNP as a fixed effect. As MLMA employs restricted maximum likelihood methods, the regression is conducted on the linear scale, rather than the liability scale. As such, test statistics (betas and their corresponding standard errors) were transformed to Odds Ratios (ORs) and their corresponding 95% Confidence Intervals (CI) on the liability scale using a Taylor transformation expansion series(20, 21). Sample prevalence estimates as described in Supplementary Table 1 were used to transform observed scale betas and standard errors to liability scale odds ratios and confidence intervals. The odds ratio and confidence intervals were derived as follows, where P = disease prevalence, β = observed scale beta, SE = observed scale standard error, OR = liability scale odds ratio, CI = liability scale confidence intervals:

OR = OR_numerator_/OR_denominator_

Where OR_numerator_ = (P + β)/(1 - P - β)

and OR_denominator_ = P/(1-P)

and CI = ((P+(β±(1.96*SE))/(1-P-(P+(β±(1.96*SE))))/OR_denominator_

# Pathway and functional genomic analyses

## Pathway analysis: DEPICT

Gene sets were analyzed using Data-driven Expression Prioritized Integration for Complex Traits (DEPICT) (https://github.com/perslab/depict)(22) to (i) prioritize genes in independent loci, (ii) identify reconstituted gene sets enriched by genes selected, which may represent biologically relevant pathways and systems, and (iii) determine enriched tissue/cell types.

SNPs with a meta-analysis P-value of P≤1x10-5 were subjected to clump-based linkage disequilibrium pruning using PLINK(23) (LD r^2^ > 0.1; physical kb threshold = 500kb; 1000 Genomes Project Phase 1 CEU, GBR, TSI genotype data(24)) to create a set of approximately independent “lead SNPs”. Associated regions were defined by linkage disequilibrium (LD) around the “lead SNPs” (LD r^2^ > 0.5; 1000 Genomes Project Phase 1 CEU, GBR, TSI genotype data) and genes selected where they mapped within or overlapping the regions identified (genome build GRCh37). Genes within the high-LD HLA locus (chr6:25000000-35000000) were removed and overlapping regions merged. If no gene was present in a region, the nearest gene was selected.

DEPICT is based on predicted function of genes derived using the results of 77,840 microarrays from two human, one rat and one mouse Affymetrix gene expression platforms from the Omnibus (GeO) database(25), each covering expression of 19,997 genes. These microarrays were renormalized before computing all pairwise correlations (Pearson’s) on a probe-by-probe correlation matrix for each platform. PCs analyses were performed within each of the four correlation matrices, retaining 777 and 377 eigenvectors (termed transcriptional components, TCs) from the human platforms, and 677 and 375 TCs from the mouse and rat platforms, respectively. All human genes were mapped to Ensembl identifiers(26) (mouse and rat genes converted to human homologous using Ensembl database orthology mapping). The loadings of each gene onto each TC form a gene-TC matrix (19,997 gene rows x 2,206 TC columns) used to predict gene function across 14,461 reconstituted gene sets from a wide spectrum of biological annotations (i.e. Gene Ontology(27), Kyopto Encyclopedia of Genes and Genomes(28), REACTOME(29), InWeb database(30) and The Mouse Genetics initiative(31); only gene sets with between 10 and 500 were included).

For each gene set, enrichment on each TC is computed using Z-scores derived from Welch’s t-test to assess whether TC loadings for genes within a gene set significantly deviate from all other genes’ loadings, resulting in a gene set-TC matrix of Z-scores (14,461 reconstituted gen set rows x 2 206 TC columns). To obtain gene function predictions, each gene’s 2,206 TC loading (from the gene-TC matrix) is correlated with the Z-score TC profile of each gene set (from the gene set-TC matrix) in order to quantify each gene’s likelihood of being part of a given gene set. Finally, correlation P-values are converted to membership Z-scores to form a gene-gene set matrix (19,997 gene rows x 14,461 reconstituted gene set columns), which is used in order to perform gene prioritization and gene set enrichment analysis.

*Gene prioritisation*

To prioritize genes, the similarity of a given gene to genes within other associated loci is quantified by a metric derived by correlating their gene’s reconstituted gene set membership
Z-scores across all 14,461 gene sets. To control for the bias introduced by gene length and gene density, each gene’s similarity score is normalized based on the distribution of a given gene’s similarity score from 1,000 sets of gene-density-matched loci obtained from 200 permuted null GWAS based on randomly distributed phenotypes. Finally, experiment-wide FDR are derived using the top SNPs from the null GWAS by repeating the scoring and bias adjustment steps 20 times. FDR is defined as the number of times the observed gene had a prioritization P-value equal or higher than genes across all 20 null GWAS divided by the rank of the observed gene in the actual data.

Although not driven by phenotype-specific hypothesis, DEPICT assumes that genes involved in the same phenotype will tend to share functional annotations and patterns of co-expression. Therefore, genes driving the association should converge on particular systems and pathways. To determine whether the genes in the associated loci share biological functions, enrichment of reconstituted gene sets is quantified by summing gene set membership Z-scores of all genes within an independently associated loci and then sum across all loci. This is repeated 1,000 times based on random loci that are matched by gene density. Then, the real Z-score is adjusted by subtracting the mean of the 1,000 Z-scores permuted and dividing by their standard deviation. Finally, the adjusted Z-score is converted to a P-value. Experiment-wide FDRs are estimated by repeating this procedure 20 times (similar to gene prioritization).

*Tissue and cell analysis*

DEPICT contains the results of 37,427 human Affymetrix HGU133a2.0 platform microarrays to determine if genes in the associated loci are highly expressed in any of the 209 Medical Subject Heading (MeSH) tissue and cell type annotations. A gene-tissue/cell type expression matrix (similar to the gene-gene set matrix) is constructed by averaging gene expression levels of microarray samples with the same MeSH annotation and normalizing across tissue/cell type annotations and across the columns of the matrix. Only tissue/cell type annotations covered by at least 10 microarrays are retained. The method to determine enrichment of a specific tissue or cell line is conceptually identical to the gene set enrichment analysis described above.

## Functional genomic analysis

For SNPs identified by LD clumping, evidence of expression quantitative trait loci (eQTL) and functional annotation were explored using publicly available online resources. The Genotype-Tissue Expression Portal (GTEx) (http://www.gtexportal.org) was used to identify eQTLs associated with the SNPs. Functional annotation was investigated using the Regulome DB database(32) (http://www.regulomedb.org/). Regulome DB was used to identify regulatory DNA elements in non-coding and intergenic regions of the genome in normal cell lines/tissues.

# Polygenic profiling analysis

# Accounting for population stratification using principal components

## GS:SFHS

The first 20 principal components (PCs) were derived using GCTA software(17), incorporating data from all autosomal SNPs (n=561,125) and all genotyped individuals who passed quality control (n=18,725). To determine the number of PCs required, linear regression models of the trait onto up to 20 PCs were run for MDD, rMDD, fMDD and mMDD. PCs are included in the model in their order of importance based on their variance explained in the kinship matrix. The best fitting model for each trait was evaluated using a stepwise approach, adding one PC at a time, and using a likelihood-ratio test (LRT), the output of which was assessed against a mixed 0.5(χ^2^)+0.5(0) distribution(33). Significant PCs for each trait are shown in Supplementary Table 2. Visualizations of log-likelihood and residual variance from the linear regression models are shown in Supplementary Figure 1, indicating that the first 20 PCs ensure adequate correction for population stratification in MDD, rMDD and fMDD. No PC improved model fit for mMDD, however, for continuity 20 PCs were incorporated into subsequent models across all traits.

| **Trait** | **PCs which significantly improved model fit** |
| --- | --- |
| MDD | 3, 15, 16, 18, 19 |
| rMDD | 15, 18, 19 |
| fMDD | 15, 16, 19, 20 |
| mMDD | - |

Supplementary Table 2. Principal components which significantly improve model fit (at p<0.05) for definitions of MDD.


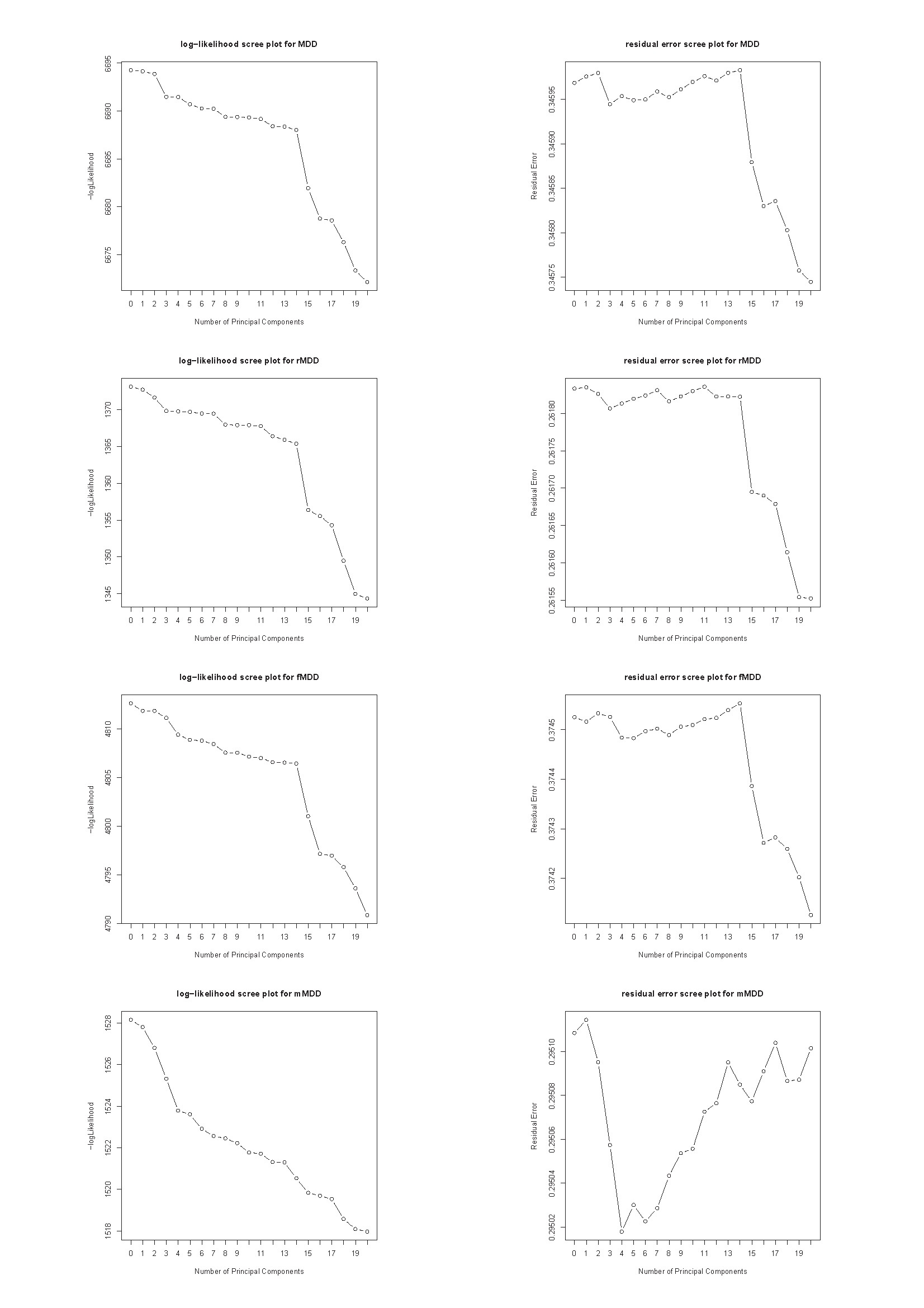


Supplementary Figure 1. Scree plots of the log likelihood and residual error from regressing up to 20 PCs onto MDD, rMDD, fMDD and mMDD.

# Accounting for shared environmental factors in GS:SFHS

## Modelling shared household environment

Household environment was modelled based on parent-offspring relationships. Two household environment variables were created, representing historic and recent shared environment. This was to avoid inferring a common environment between grandparents and grandchildren in three generation families, where the individuals in the mid-parent generation are parents and offspring concurrently. Three generation families were split into historic (grandparent - mid-parent) and recent (mid-parent - offspring) groups. Two generation families were then assigned to one of these groups. To establish which group each two generation family should be assigned to, an optimal age cut-off was calculated. To determine this value, three generation (n=721) and two generation (n=8 798) families were identified and the age range of the parents in these groups visualized, as shown in Supplementary Figure 2. The mean age for each group was 74 (grandparents from three generation families), 49 (mid-parents from three generation families) and 60 (parents from two generation families), respectively.


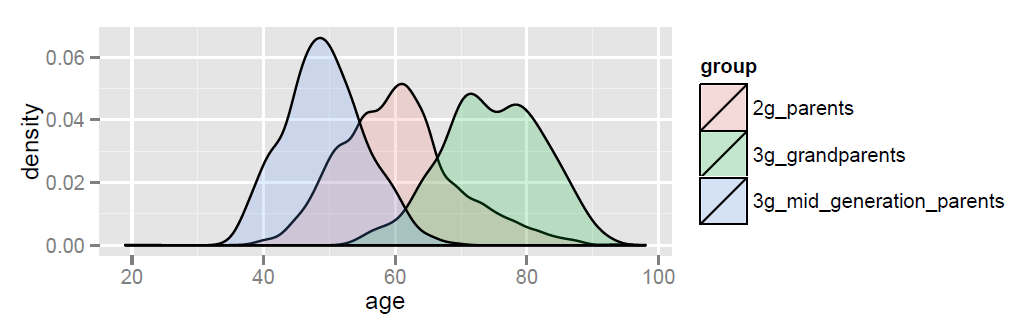


Supplementary Figure 2. Age distributions for parents and mid-parents from two and three generation families.

All possible age cut-offs in this range (49 - 74 years) were tested, using the average age of each parent couple to ensure that parents belonged to the same generational group. Using age 59 as the age cut-off minimized the variance between the two groups, and resulted in approximately equal group numbers. Parents and offspring were then assigned their own unique identifier for either the historic or recent shared environment. In analyses that fitted common household environment as a random effect, both old and young household were fitted simultaneously. This is because the two household environmental variables were generated for the purpose of more accurately modelling parent-offspring environmental effects in multi-generational families, rather than specifically seeking to model these generations separately for hypothetical purposes.

## Modelling shared sibling environment

A shared sibling environment was calculated as the effect of common parents, whereby individuals who shared two parents were assigned a same unique identifier, reflecting the shared environment of full-sibs.

## Modelling shared spouse environment

A shared spousal environment was determined by coding individuals with a common child as having an unique identifier.

Individuals who did not share a common environment variable with any other participants were assigned their own unique identifier, demonstrating that they had a common household, sib and/or spouse environment with themselves. These environmental variables do not account for more nuanced modelling of shared environments (e.g. half-sib, grandparent, maternal or paternal effects), however they serve the purpose of broadly accounting for shared environment so as not to overestimate genetic effects.

## Shared environment model selection

The best fitting model for each trait was identified using a stepwise approach by comparing null and alternative models using a likelihood-ratio test (LRT), the output of which was assessed against a mixed 0.5(χ^2^)+0.5(0) distribution(33). The initial null model included only an additive genetic effect (ped) as a random effect. Three alternative models were generated, each fitting an individual environmental variable as an additional random effect (e.g. ped + household, ped + sibling, ped + spouse). Usually additional environmental effects would be added until all four random effects were incorporated into the model, however after fitting each environmental effect individually it became apparent that no environmental effect improved model fit relative to the null model after multiple testing correction, therefore the stepwise method was stopped at this point. Model selection was implemented in ASReml-R, with MDD subtype as the dependent variable and the first 20 PCs fitted as fixed effects in all models. Detailed results tables from the likelihood ratio test for each stage of this stepwise model fitting approach are shows in Supplementary Table 3.

| **Trait** | **Model** | **Constraint** | **Null** | **Full** | **χ2** | **P-value** |
| --- | --- | --- | --- | --- | --- | --- |
| MDD | ped + household | Positive | 10590.96 | 10591.65 | 0.69 | 0.41 |
| rMDD |  | Positive | 14693.92 | 14694.51 | 0.60 | 0.44 |
| fMDD |  | Boundary | 5364.86 | 5364.86 | 0 | 1 |
| mMDD |  | Boundary | 5550.64 | 5550.64 | 0 | 1 |
| MDD | ped + sibling | Boundary | 10590.96 | 10590.96 | 0 | 1 |
| rMDD |  | Boundary | 14693.92 | 14693.92 | 0 | 1 |
| fMDD |  | Boundary | 5364.86 | 5364.86 | 0 | 1 |
| mMDD |  | Boundary | 5550.64 | 5550.64 | 0 | 1 |
| MDD | ped + spouse | Positive | 10590.96 | 10595.21 | 4.26 | 0.04 |
| rMDD |  | Positive | 14693.92 | 14697.93 | 4.02 | 0.04 |
| fMDD |  | *Singular* | *5364.86* | *-* | *-* | *-* |
| mMDD |  | *Singular* | *5550.64* | *-* | *-* | *-* |

Supplementary Table 3. Likelihood ratio test results with the null model fitting ped as a random effect, and the alternative model fitting ped and shared spouse environment as random effects. After multiple testing correction, the alternative model did not significantly improve model fit for any trait. Traits for which the alternative model was erroneous are highlighted in italics.

# Supplementary Results

# Genome wide association analysis


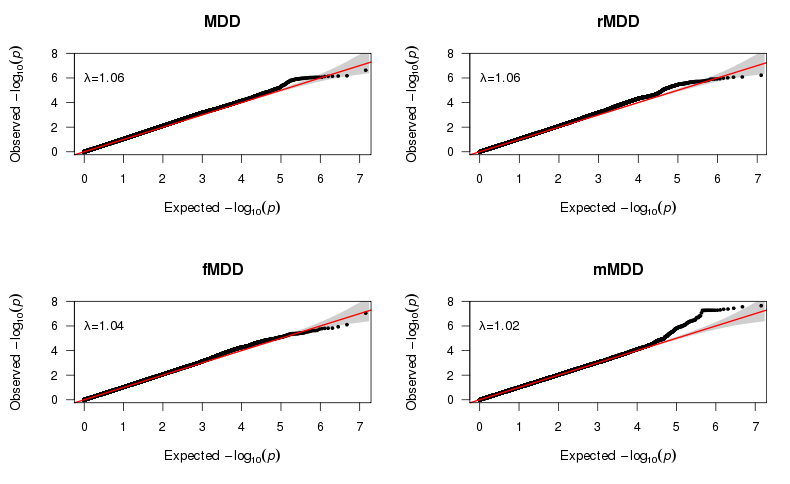


Supplementary Figure 3. Quantile-quantile plot for meta-analysis of MDD, rMDD, fMDD and mMDD, showing expected distribution of GWAS test statistics, -log10(p), versus the observed distribution. The ratio of the observed median χ^2^ to that expected by chance, denoted λ, is indicated in the top left hand corner.

##

## Meta-analysis of MDD

| **SNP** | **CHR** | **POS** | **A1/A2** | **β (SE)** | **P** | **Direction** | **Genes** |
| --- | --- | --- | --- | --- | --- | --- | --- |
| rs4435501 | 2 | 96981369 | A/C | 0.13 (0.03) | 4.24x10^-6^ | ++ | *ITPRIPL1* |
| rs1874256 | 3 | 28994183 | A/G | 0.07 (0.02) | 9.04x10^-6^ | ++ | - |
| rs73063271 | 3 | 38467249 | A/G | -0.17 (0.04) | 5.98x10^-6^ | -- | *XYLB* |
| rs73249855 | 4 | 26764902 | A/G | 0.21 (0.05) | 9.95x10^-6^ | ++ | *TBCD19* |
| rs11099341 | 4 | 136328202 | A/G | -0.09 (0.02) | 7.42x10^-6^ | -- | - |
| rs342318 | 4 | 157788955 | A/G | 0.10 (0.02) | 6.37x10^-6^ | ++ | *PDGFC* |
| rs115981586 | 4 | 161694897 | A/G | 0.21 (0.05) | 7.70x10^-6^ | ++ | - |
| rs56390503 | 4 | 187552576 | T/C | 0.13 (0.03) | 9.08x10^-7^ | ++ | *FAT1* |
| rs2964802 | 5 | 10820843 | T/C | 0.09 (0.02) | 6.73x10^-7^ | ++ | - |
| rs11743328 | 5 | 112015555 | T/C | -0.10 (0.02) | 6.55x10^-6^ | -- | *LOC102467216* |
| rs144735131 | 6 | 26121278 | A/C | -0.40 (0.08) | 1.91x10^-6^ | -- | *HIST1H2BC/HIST1A2AC* |
| rs9648182 | 7 | 13794849 | A/T | -0.11 (0.03) | 7.54x10^-6^ | -- | - |
| rs117715236 | 8 | 32457970 | A/T | -0.24 (0.05) | 4.06x10^-6^ | -- | *NRG1* |
| rs12547341 | 8 | 92702518 | A/G | 0.08 (0.02) | 4.50x10^-6^ | ++ | - |
| rs145478122 | 8 | 96448589 | A/G | -0.30 (0.07) | 8.70x10^-6^ | -- | *C8orf37-AS1* |
| rs10959631 | 9 | 11220986 | T/C | -0.10 (0.02) | 4.34x10^-6^ | -- | - |
| rs12240686 | 10 | 118638182 | A/G | -0.18 (0.04) | 6.26x10^-6^ | -- | *ENO4* |
| rs11033303 | 11 | 35871266 | A/G | 0.09 (0.02) | 2.37x10^-7^ | ++ | - |
| rs80208956 | 11 | 69497269 | A/G | 0.30 (0.07) | 5.69x10^-6^ | ++ | *ORA0V1* |
| rs10736455 | 11 | 111064723 | A/C | -0.16 (0.04) | 3.67x10^-6^ | -- | *-* |
| rs8050755 | 16 | 2119187 | T/C | 0.11 (0.02) | 9.48x10^-6^ | ++ | *TSC2* |
| rs35888008 | 16 | 49477272 | A/T | 0.09 (0.02) | 3.65x10^-6^ | ++ | *-* |
| rs140555 | 22 | 45415897 | T/C | -0.09 (0.02) | 2.38x10^-6^ | -- | *PHF21B* |

Supplementary Table 4. Linkage disequilibrium (LD)-independent associations for depression (sorted by genomic position according to UCSC hg19/NCBI Build 37) with association P value (P) ≤ 1x10^-5^. Column A1/A2 contains the reference and alternate alleles for the index SNP, respectively. The meta-analysis regression coefficient (β) column pertains to the reference allele (A1). Chr and Position denote the location of the index SNP. SE is the standard error for β. The direction of effect of the index SNP in GS:SFHS and UKB is shown in the Direction column. The final column, Genes, indicates protein-coding reference sequence genes within 10kb of the associated loci.

| **SNP** | **CHR** | **POS** | **GS:SFHS** | | | |  | **UKB** | | |
| --- | --- | --- | --- | --- | --- | --- | --- | --- | --- | --- |
|  |  |  | **β** | **SE** | **P** | **Freq** | **β** | **SE** | **P** | **Freq** |
| rs4435501 | 2 | 96981369 | -0.11 | 0.05 | 0.04 | 0.10 | -0.14 | 0.03 | 3.45x10^-5^ | 0.10 |
| rs1874256 | 3 | 28994183 | 0.08 | 0.03 | 0.01 | 0.39 | 0.07 | 0.02 | 0.0002 | 0.38 |
| rs73063271 | 3 | 38467249 | 0.22 | 0.07 | 0.001 | 0.05 | 0.15 | 0.05 | 0.001 | 0.04 |
| rs73249855 | 4 | 26764902 | 0.21 | 0.09 | 0.02 | 0.03 | 0.21 | 0.06 | 0.0002 | 0.03 |
| rs11099341 | 4 | 136328202 | -0.07 | 0.04 | 0.08 | 0.20 | -0.10 | 0.02 | 3.01x10^-5^ | 0.20 |
| rs342318 | 4 | 157788955 | 0.07 | 0.04 | 0.09 | 0.16 | 0.11 | 0.03 | 1.92x10^-5^ | 0.16 |
| rs115981586 | 4 | 161694897 | -0.24 | 0.09 | 0.006 | 0.04 | -0.20 | 0.06 | 0.0004 | 0.04 |
| rs56390503 | 4 | 187552576 | 0.09 | 0.05 | 0.06 | 0.10 | 0.14 | 0.03 | 3.96x10^-6^ | 0.11 |
| rs2964802 | 5 | 10820843 | -0.09 | 0.04 | 0.01 | 0.27 | -0.09 | 0.02 | 2.18x10^-5^ | 0.28 |
| rs11743328 | 5 | 112015555 | 0.12 | 0.04 | 0.002 | 0.17 | 0.09 | 0.03 | 0.0009 | 0.17 |
| rs144735131 | 6 | 26121278 | -0.38 | 0.16 | 0.02 | 0.01 | -0.40 | 0.10 | 3.19x10^-5^ | 0.02 |
| rs9648182 | 7 | 13794849 | 0.18 | 0.04 | 1.52x10^-5^ | 0.13 | 0.07 | 0.03 | 0.02 | 0.12 |
| rs117715236 | 8 | 32457970 | -0.32 | 0.09 | 0.0005 | 0.04 | -0.21 | 0.06 | 0.001 | 0.03 |
| rs12547341 | 8 | 92702518 | -0.08 | 0.03 | 0.02 | 0.40 | -0.08 | 0.02 | 8.83x10^-5^ | 0.38 |
| rs145478122 | 8 | 96448589 | 0.41 | 0.11 | 0.0003 | 0.01 | 0.24 | 0.08 | 0.004 | 0.01 |
| rs10959631 | 9 | 11220986 | -0.08 | 0.04 | 0.04 | 0.21 | -0.10 | 0.02 | 3.76x10^-5^ | 0.20 |
| rs12240686 | 10 | 118638182 | 0.17 | 0.07 | 0.02 | 0.04 | 0.19 | 0.05 | 8.60x10^-5^ | 0.04 |
| rs11033303 | 11 | 35871266 | 0.09 | 0.03 | 0.002 | 0.38 | 0.09 | 0.02 | 3.27x10^-5^ | 0.37 |
| rs80208956 | 11 | 69497269 | 0.36 | 0.12 | 0.003 | 0.01 | 0.27 | 0.08 | 0.0006 | 0.01 |
| rs10736455 | 11 | 111064723 | -0.11 | 0.07 | 0.11 | 0.06 | -0.19 | 0.04 | 8.62x10^-6^ | 0.06 |
| rs8050755 | 16 | 2119187 | -0.11 | 0.05 | 0.02 | 0.14 | -0.11 | 0.03 | 0.0002 | 0.13 |
| rs35888008 | 16 | 49477272 | -0.11 | 0.04 | 0.005 | 0.22 | -0.09 | 0.02 | 0.0002 | 0.23 |
| rs140555 | 22 | 45415897 | -0.10 | 0.04 | 0.009 | 0.25 | -0.09 | 0.02 | 8.69x10^-5^ | 0.26 |

Supplementary Table 5. Association results from GWAS of depression for index SNPs with P≤1x10^-5^ in GS:SFHS and UKB datasets separately.

## Meta-analysis of recurrent MDD

| **SNP** | **CHR** | **POS** | **A1/A2** | **β (SE)** | **P** | **Direction** | **Genes** |
| --- | --- | --- | --- | --- | --- | --- | --- |
| rs114508355 | 1 | 109500243 | C/G | -0.46 (0.10) | 9.40x10^-6^ | -- | *CLCC1* |
| rs13011737 | 2 | 40066622 | A/G | 0.10 (0.02) | 1.23x10^-6^ | ++ | - |
| rs288342 | 2 | 183665207 | T/C | -0.09 (0.02) | 7.29x10^-6^ | -- | - |
| rs507385 | 3 | 7010717 | A/T | -0.13 (0.03) | 8.52x10^-6^ | -- | *GRM7* |
| rs2291479 | 3 | 178174944 | A/C | -0.10 (0.02) | 9.65x10^-7^ | -- | - |
| rs2964802 | 5 | 10820843 | T/C | 0.10 (0.02) | 1.95x10^-6^ | ++ | - |
| rs114927772 | 6 | 10818929 | A/T | 0.35 (0.07) | 2.89x10^-6^ | ++ | *MAK* |
| rs117901488 | 6 | 160603132 | T/C | 0.20 (0.04) | 5.73x10^-6^ | ++ | - |
| rs7835340 | 8 | 67052569 | A/G | 0.09 (0.02) | 3.19x10^-6^ | ++ | *TRIM55* |
| rs79802045 | 8 | 101349133 | A/G | -0.25 (0.06) | 4.90x10^-6^ | -- | *RNF19A* |
| rs10959631 | 9 | 11220986 | T/C | -0.12 (0.02) | 8.34x10^-7^ | -- | - |
| rs2460546 | 10 | 43736574 | A/G | 0.29 (0.06) | 3.81x10^-6^ | ++ | *RASGEF1A* |
| rs11033303 | 11 | 35871266 | A/G | 0.11 (0.02) | 6.02x10^-7^ | ++ | - |
| rs17615103 | 11 | 60238405 | T/C | 0.22 (0.05) | 6.48x10^-6^ | ++ | *MS4A1* |
| rs1031396 | 13 | 91559812 | T/G | -0.40 (0.09) | 4.67x10^-6^ | -- | *LINC00410* |
| rs9300648 | 13 | 101759340 | T/C | -0.16 (0.03) | 5.72x10^-6^ | -- | *NALCN* |
| rs4438172 | 13 | 111448658 | A/T | 0.11 (0.02) | 8.72x10^-7^ | ++ | - |
| rs60716536 | 14 | 97623218 | A/G | -0.13 (0.03) | 3.09x10^-6^ | -- | *-* |
| rs1486437 | 16 | 51899860 | A/G | -0.13 (0.03) | 9.46x10^-6^ | -- | - |
| rs1792737 | 18 | 53846367 | C/G | -0.10 (0.02) | 2.25x10^-6^ | -- | - |
| rs4941058 | 18 | 59448225 | C/G | -0.13 (0.03) | 1.20x10^-6^ | -- | - |
| rs8140097 | 22 | 19537262 | T/C | 0.12 (0.02) | 1.96x10^-6^ | ++ | - |
| rs5760842 | 22 | 25482991 | A/G | -0.09 (0.02) | 2.19x10^-6^ | -- | *-* |

Supplementary Table 6. LD-independent associations for recurrent depression (sorted by genomic position according to UCSC hg19/NCBI Build 37) with association P value (P) ≤ 1x10^-5^. Column A1/A2 contains the reference and alternate alleles for the index SNP, respectively. The meta-analysis regression coefficient (β) column pertains to the reference allele (A1). Chr and Position denote the location of the index SNP. SE is the standard error for β. The direction of effect of the index SNP in GS:SFHS and UKB is shown in the Direction column. The final column, Genes, indicates protein-coding reference sequence genes within 10kb of the associated loci.

| **SNP** | **CHR** | **POS** | **GS:SFHS** | | | | **UKB** | | | |
| --- | --- | --- | --- | --- | --- | --- | --- | --- | --- | --- |
|  |  |  | **β** | **SE** | **P** | **Freq** | **β** | **SE** | **P** | **Freq** |
| rs114508355 | 1 | 109500243 | -0.43 | 0.21 | 0.04 | 0.01 | -0.47 | 0.12 | 8.81E-05 | 0.01 |
| rs13011737 | 2 | 40066622 | 0.11 | 0.04 | 0.006 | 0.48 | 0.10 | 0.02 | 5.50E-05 | 0.48 |
| rs288342 | 2 | 183665207 | 0.10 | 0.04 | 0.02 | 0.38 | 0.08 | 0.02 | 0.0001 | 0.40 |
| rs507385 | 3 | 7010717 | 0.15 | 0.06 | 0.02 | 0.11 | 0.12 | 0.03 | 0.0002 | 0.12 |
| rs2291479 | 3 | 178174944 | 0.06 | 0.04 | 0.16 | 0.41 | 0.11 | 0.02 | 1.37x10^-6^ | 0.39 |
| rs2964802 | 5 | 10820843 | -0.13 | 0.05 | 0.008 | 0.27 | -0.10 | 0.02 | 6.01x10^-5^ | 0.28 |
| rs114927772 | 6 | 10818929 | -0.12 | 0.17 | 0.47 | 0.02 | -0.40 | 0.08 | 1.23x10^-6^ | 0.02 |
| rs117901488 | 6 | 160603132 | 0.09 | 0.11 | 0.37 | 0.04 | 0.22 | 0.05 | 4.48x10^-6^ | 0.05 |
| rs7835340 | 8 | 67052569 | -0.09 | 0.05 | 0.05 | 0.41 | -0.10 | 0.02 | 2.30x10^-5^ | 0.40 |
| rs79802045 | 8 | 101349133 | -0.29 | 0.12 | 0.02 | 0.04 | -0.24 | 0.06 | 9.09x10^-5^ | 0.03 |
| rs10959631 | 9 | 11220986 | -0.11 | 0.05 | 0.04 | 0.21 | -0.12 | 0.03 | 8.00x10^-6^ | 0.20 |
| rs2460546 | 10 | 43736574 | 0.36 | 0.12 | 0.003 | 0.02 | 0.26 | 0.07 | 0.0003 | 0.02 |
| rs11033303 | 11 | 35871266 | 0.08 | 0.04 | 0.07 | 0.38 | 0.12 | 0.02 | 2.25x10^-6^ | 0.37 |
| rs17615103 | 11 | 60238405 | 0.28 | 0.11 | 0.01 | 0.03 | 0.21 | 0.05 | 0.0002 | 0.04 |
| rs1031396 | 13 | 91559812 | 0.23 | 0.17 | 0.18 | 0.01 | 0.45 | 0.10 | 6.04x10^-6^ | 0.01 |
| rs9300648 | 13 | 101759340 | 0.14 | 0.08 | 0.08 | 0.07 | 0.16 | 0.04 | 2.71x10^-5^ | 0.08 |
| rs4438172 | 13 | 111448658 | -0.09 | 0.05 | 0.07 | 0.25 | -0.11 | 0.03 | 4.38x10^-6^ | 0.25 |
| rs60716536 | 14 | 97623218 | -0.08 | 0.06 | 0.19 | 0.14 | -0.14 | 0.03 | 5.30x10^-6^ | 0.14 |
| rs1486437 | 16 | 51899860 | 0.19 | 0.06 | 0.002 | 0.11 | 0.11 | 0.03 | 0.0008 | 0.12 |
| rs1792737 | 18 | 53846367 | -0.11 | 0.05 | 0.02 | 0.39 | -0.09 | 0.02 | 3.79x10^-5^ | 0.36 |
| rs4941058 | 18 | 59448225 | 0.18 | 0.05 | 0.0007 | 0.17 | 0.11 | 0.03 | 0.0002 | 0.16 |
| rs8140097 | 22 | 19537262 | 0.13 | 0.05 | 0.01 | 0.17 | 0.11 | 0.03 | 5.30x10^-5^ | 0.18 |
| rs5760842 | 22 | 25482991 | 0.09 | 0.04 | 0.04 | 0.40 | 0.09 | 0.02 | 2.18x10^-5^ | 0.41 |

Supplementary Table 7. Association results from GWAS of recurrent depression for index SNPs with P≤1x10^-5^ in GS:SFHS and UKB datasets separately.

## Meta-analysis of MDD in females only

| **SNP** | **CHR** | **POS** | **A1/A2** | **β (SE)** | **P** | **Direction** | **Genes** |
| --- | --- | --- | --- | --- | --- | --- | --- |
| rs146951131 | 1 | 107008293 | T/G | 0.36 (0.08) | 9.91x10^-6^ | ++ | *-* |
| rs12464040 | 2 | 84855612 | T/G | 0.15 (0.03) | 1.65x10^-6^ | ++ | *DNAH6* |
| rs1874256 | 3 | 28994183 | A/G | 0.10 (0.02) | 4.17x10^-6^ | ++ | *RBMS3* |
| rs116569570 | 4 | 72816089 | A/G | -0.16 (0.04) | 5.33x10^-6^ | -- | *-* |
| rs187279821 | 4 | 128757383 | A/G | -0.42 (0.09) | 4.70x10^-6^ | -- | *HSPA4L* |
| rs6893200 | 5 | 10806757 | T/C | -0.11 (0.02) | 4.98x10^-6^ | -- | *-* |
| rs111404313 | 6 | 90568830 | A/T | -0.32 (0.07) | 3.75x10^-6^ | -- | *CASP8AP2* |
| rs9648182 | 7 | 13794849 | A/T | -0.17 (0.03) | 9.14x10^-8^ | -- | *-* |
| rs10227749 | 7 | 14443892 | T/C | 0.12 (0.03) | 3.93x10^-6^ | ++ | *DGKB* |
| rs17176546 | 7 | 81880914 | A/G | 0.26 (0.05) | 7.93x10^-7^ | ++ | *CACNA2D1* |
| rs79936639 | 7 | 152287759 | A/G | -0.17 (0.04) | 9.64x10^-6^ | -- | *-* |
| rs79225303 | 8 | 141129836 | T/G | -0.17 (0.04) | 2.37x10^-6^ | -- | *TRAPPC9* |
| rs61842316 | 10 | 16753720 | T/C | 0.27 (0.06) | 2.94x10^-6^ | ++ | *RSU1* |
| rs11613048 | 12 | 30353975 | A/G | -0.10 (0.02) | 7.75x10^-6^ | -- | *-* |
| rs1448094 | 12 | 86343135 | T/G | 0.10 (0.02) | 1.18x10^-6^ | ++ | *-* |
| rs73228161 | 12 | 94662671 | A/G | 0.19 (0.04) | 5.84x10^-6^ | ++ | *PLXNC1* |
| rs139073054 | 13 | 87651258 | T/C | 0.35 (0.07) | 2.38x10^-6^ | ++ | *-* |
| rs79306025 | 14 | 85598792 | A/T | 0.44 (0.09) | 1.82x10^-6^ | ++ | *-* |
| rs17705482 | 15 | 36835788 | T/C | -0.11 (0.03) | 9.09x10^-6^ | -- | *-* |
| rs74031748 | 15 | 98494154 | A/C | -0.19 (0.04) | 6.85x10^-6^ | -- | *-* |
| rs35888008 | 16 | 49477272 | A/T | 0.12 (0.03) | 6.16x10^-6^ | ++ | *-* |

Supplementary Table 8. LD-independent associations for depression in females only (sorted by genomic position according to UCSC hg19/NCBI Build 37) with association P value (P) ≤ 1x10^-5^. Column A1/A2 contains the reference and alternate alleles for the index SNP, respectively. The meta-analysis regression coefficient (β) column pertains to the reference allele (A1). Chr and Position denote the location of the index SNP. SE is the standard error for β. The direction of effect of the index SNP in GS:SFHS and UKB is shown in the Direction column. The final column, Genes, indicates protein-coding reference sequence genes within 10kb of the associated loci.

| **SNP** | **CHR** | **POS** | **GS:SFHS** | | | |  | **UKB** | | |
| --- | --- | --- | --- | --- | --- | --- | --- | --- | --- | --- |
|  |  |  | **β** | **SE** | **P** | **Freq** | **β** | **SE** | **P** | **Freq** |
| rs146951131 | 1 | 107008293 | 0.48 | 0.13 | 0.0002 | 0.01 | 0.28 | 0.10 | 0.006 | 0.02 |
| rs12464040 | 2 | 84855612 | 0.10 | 0.05 | 0.05 | 0.13 | 0.17 | 0.04 | 6.35x10^-6^ | 0.14 |
| rs1874256 | 3 | 28994183 | 0.10 | 0.04 | 0.005 | 0.39 | 0.10 | 0.03 | 0.0002 | 0.38 |
| rs116569570 | 4 | 72816089 | 0.24 | 0.06 | 5.15x10^-5^ | 0.09 | 0.12 | 0.05 | 0.009 | 0.09 |
| rs187279821 | 4 | 128757383 | 0.57 | 0.13 | 1.61x10^-5^ | 0.01 | 0.28 | 0.12 | 0.03 | 0.01 |
| rs6893200 | 5 | 10806757 | -0.08 | 0.04 | 0.06 | 0.27 | -0.13 | 0.03 | 2.30x10^-5^ | 0.28 |
| rs111404313 | 6 | 90568830 | 0.31 | 0.11 | 0.004 | 0.03 | 0.33 | 0.09 | 0.0003 | 0.02 |
| rs9648182 | 7 | 13794849 | 0.27 | 0.05 | 4.88x10^-8^ | 0.13 | 0.10 | 0.04 | 0.02 | 0.12 |
| rs10227749 | 7 | 14443892 | -0.10 | 0.04 | 0.03 | 0.24 | -0.13 | 0.03 | 3.49x10^-5^ | 0.24 |
| rs17176546 | 7 | 81880914 | 0.15 | 0.08 | 0.06 | 0.05 | 0.34 | 0.07 | 9.40x10^-7^ | 0.04 |
| rs79936639 | 7 | 152287759 | -0.20 | 0.07 | 0.003 | 0.09 | -0.15 | 0.05 | 0.0008 | 0.09 |
| rs79225303 | 8 | 141129836 | -0.19 | 0.06 | 0.002 | 0.12 | -0.16 | 0.04 | 0.0003 | 0.11 |
| rs61842316 | 10 | 16753720 | -0.17 | 0.09 | 0.06 | 0.05 | -0.34 | 0.08 | 6.48x10^-6^ | 0.04 |
| rs11613048 | 12 | 30353975 | -0.12 | 0.04 | 0.003 | 0.44 | -0.09 | 0.03 | 0.0008 | 0.45 |
| rs1448094 | 12 | 86343135 | 0.09 | 0.04 | 0.01 | 0.43 | 0.11 | 0.03 | 2.68x10^-5^ | 0.44 |
| rs73228161 | 12 | 94662671 | 0.26 | 0.07 | 0.0001 | 0.07 | 0.14 | 0.05 | 0.006 | 0.07 |
| rs139073054 | 13 | 87651258 | 0.32 | 0.11 | 0.005 | 0.02 | 0.37 | 0.10 | 0.0001 | 0.02 |
| rs79306025 | 14 | 85598792 | -0.28 | 0.17 | 0.11 | 0.01 | -0.50 | 0.11 | 3.88x10^-6^ | 0.02 |
| rs17705482 | 15 | 36835788 | 0.14 | 0.04 | 0.001 | 0.22 | 0.10 | 0.03 | 0.002 | 0.22 |
| rs74031748 | 15 | 98494154 | 0.17 | 0.07 | 0.008 | 0.08 | 0.19 | 0.05 | 0.0003 | 0.07 |
| rs35888008 | 16 | 49477272 | -0.12 | 0.05 | 0.008 | 0.22 | -0.12 | 0.03 | 0.0002 | 0.23 |

Supplementary Table 9. Association results from GWAS of depression in females only for index SNPs with P≤1x10^-5^ in GS:SFHS and UKB datasets separately.

## Meta-analysis of MDD in males only

| **SNP** | **CHR** | **POS** | **A1/A2** | **β (SE)** | **P** | **Direction** | **Genes** |
| --- | --- | --- | --- | --- | --- | --- | --- |
| rs41287894 | 1 | 48627454 | T/C | 0.45 (0.10) | 3.09x10-6 | ++ | *SKINT1L* |
| rs115736167 | 1 | 155266609 | C/G | -0.46 (0.09) | 1.54x10-7 | -- | *PKLR* |
| rs4478037 | 3 | 33160407 | A/G | 0.29 (0.05) | 2.29x10-8 | ++ | *CRTAP* |
| rs40493 | 3 | 61974949 | C/G | 0.13 (0.03) | 4.02x10-6 | ++ | *PTPRG* |
| rs73108923 | 3 | 63325580 | A/G | -0.30 (0.07) | 5.80x10-6 | -- | *SYNPR* |
| rs13127214 | 4 | 47280556 | C/G | 0.19 (0.04) | 6.99x10-6 | ++ | *GABRB1* |
| rs6822806 | 4 | 96298942 | T/C | -0.18 (0.04) | 4.84x10-6 | -- | *UNC5C* |
| rs116233998 | 4 | 116134817 | A/G | -0.47 (0.10) | 7.35x10-6 | -- | *-* |
| rs255428 | 5 | 62846889 | A/C | 0.13 (0.03) | 2.75x10-6 | ++ | *-* |
| rs116078874 | 6 | 38652425 | A/G | 0.47 (0.10) | 3.51x10-6 | ++ | *GLO1* |
| rs783790 | 8 | 85356742 | A/G | 0.24 (0.05) | 9.72x10-6 | ++ | *RALYL* |
| rs10758202 | 9 | 33420008 | A/G | 0.14 (0.03) | 1.82x10-6 | ++ | *-* |
| rs78245978 | 10 | 2606976 | T/G | 0.43 (0.09) | 6.02x10-6 | -+ | *-* |
| rs117778141 | 10 | 57656128 | T/C | -0.50 (0.10) | 1.38x10-6 | -- | *-* |
| rs150439352 | 11 | 60291504 | A/G | 0.47 (0.10) | 4.20x10-6 | ++ | *MS4A13* |
| rs113485090 | 11 | 73572495 | A/G | -0.32 (0.06) | 2.05x10-7 | -- | *MRPL48* |
| rs666723 | 11 | 104826604 | A/G | 0.12 (0.03) | 6.67x10-6 | ++ | *CASP4* |
| rs1380551 | 15 | 24124704 | A/G | -0.18 (0.04) | 3.96x10-7 | -- | *-* |
| rs111484992 | 15 | 101746074 | A/G | 0.16 (0.04) | 9.92x10-6 | ++ | *-* |
| rs74002781 | 16 | 2146288 | T/C | -0.21 (0.05) | 7.30x10-6 | -- | *PKD1* |
| rs117621296 | 19 | 8482184 | T/C | -0.31 (0.07) | 9.41x10-6 | -- | *Mar-02* |

Supplementary Table 10. LD-independent associations for depression in males only (sorted by genomic position according to UCSC hg19/NCBI Build 37) with association P value (P) ≤ 1x10^-5^. Column A1/A2 contains the reference and alternate alleles for the index SNP, respectively. The meta-analysis regression coefficient (β) column pertains to the reference allele (A1). Chr and Position denote the location of the index SNP. SE is the standard error for β. The direction of effect of the index SNP in GS:SFHS and UKB is shown in the Direction column. The final column, Genes, indicates protein-coding reference sequence genes within 10kb of the associated loci.

| **SNP** | **CHR** | **POS** |  | **GS:SFHS** | | |  | **UKB** | | |
| --- | --- | --- | --- | --- | --- | --- | --- | --- | --- | --- |
|  |  |  | **β** | **SE** | **P** | **Freq** | **β** | **SE** | **P** | **Freq** |
| rs41287894 | 1 | 48627454 | 0.53 | 0.20 | 0.008 | 0.01 | 0.42 | 0.11 | 0.0001 | 0.02 |
| rs115736167 | 1 | 155266609 | 0.23 | 0.18 | 0.2 | 0.02 | 0.54 | 0.10 | 1.03x10^-7^ | 0.02 |
| rs4478037 | 3 | 33160407 | -0.31 | 0.12 | 0.009 | 0.08 | -0.28 | 0.06 | 7.65x10^-7^ | 0.08 |
| rs40493 | 3 | 61974949 | 0.05 | 0.06 | 0.38 | 0.35 | 0.15 | 0.03 | 1.75x10^-6^ | 0.34 |
| rs73108923 | 3 | 63325580 | -0.27 | 0.14 | 0.06 | 0.05 | -0.31 | 0.08 | 3.62x10^-5^ | 0.05 |
| rs13127214 | 4 | 47280556 | 0.21 | 0.08 | 0.01 | 0.11 | 0.18 | 0.05 | 0.0002 | 0.10 |
| rs6822806 | 4 | 96298942 | 0.17 | 0.08 | 0.03 | 0.13 | 0.18 | 0.04 | 5.60x10^-5^ | 0.12 |
| rs116233998 | 4 | 116134817 | 0.60 | 0.20 | 0.003 | 0.01 | 0.41 | 0.12 | 0.0006 | 0.01 |
| rs255428 | 5 | 62846889 | 0.10 | 0.06 | 0.06 | 0.36 | 0.13 | 0.03 | 1.61x10^-5^ | 0.37 |
| rs116078874 | 6 | 38652425 | 0.61 | 0.17 | 0.0002 | 0.02 | 0.39 | 0.13 | 0.003 | 0.01 |
| rs783790 | 8 | 85356742 | 0.13 | 0.11 | 0.21 | 0.06 | 0.27 | 0.06 | 1.21x10^-5^ | 0.06 |
| rs10758202 | 9 | 33420008 | -0.18 | 0.06 | 0.004 | 0.32 | -0.13 | 0.03 | 9.49x10^-5^ | 0.32 |
| rs78245978 | 10 | 2606976 | -0.03 | 0.21 | 0.9 | 0.02 | 0.54 | 0.11 | 3.22x10^-7^ | 0.02 |
| rs117778141 | 10 | 57656128 | 0.40 | 0.18 | 0.02 | 0.02 | 0.56 | 0.13 | 1.56x10^-5^ | 0.01 |
| rs150439352 | 11 | 60291504 | 0.64 | 0.15 | 2.38x10^-5^ | 0.02 | 0.33 | 0.14 | 0.02 | 0.01 |
| rs113485090 | 11 | 73572495 | 0.30 | 0.11 | 0.008 | 0.05 | 0.32 | 0.07 | 8.03x10^-6^ | 0.04 |
| rs666723 | 11 | 104826604 | 0.17 | 0.05 | 0.001 | 0.42 | 0.10 | 0.03 | 0.0008 | 0.41 |
| rs1380551 | 15 | 24124704 | 0.16 | 0.08 | 0.04 | 0.14 | 0.18 | 0.04 | 3.90x10^-6^ | 0.15 |
| rs111484992 | 15 | 101746074 | 0.19 | 0.08 | 0.01 | 0.14 | 0.15 | 0.04 | 0.0003 | 0.14 |
| rs74002781 | 16 | 2146288 | -0.27 | 0.11 | 0.01 | 0.10 | -0.19 | 0.05 | 0.0002 | 0.10 |
| rs117621296 | 19 | 8482184 | 0.21 | 0.15 | 0.16 | 0.03 | 0.33 | 0.08 | 2.05x10^-5^ | 0.03 |

Supplementary Table 11. Association results from GWAS of depression in males only for index SNPs with P≤1x10^-5^ in GS:SFHS and UKB datasets separately.

## Look-up of rs4778037 in GWAS of other MDD subsets


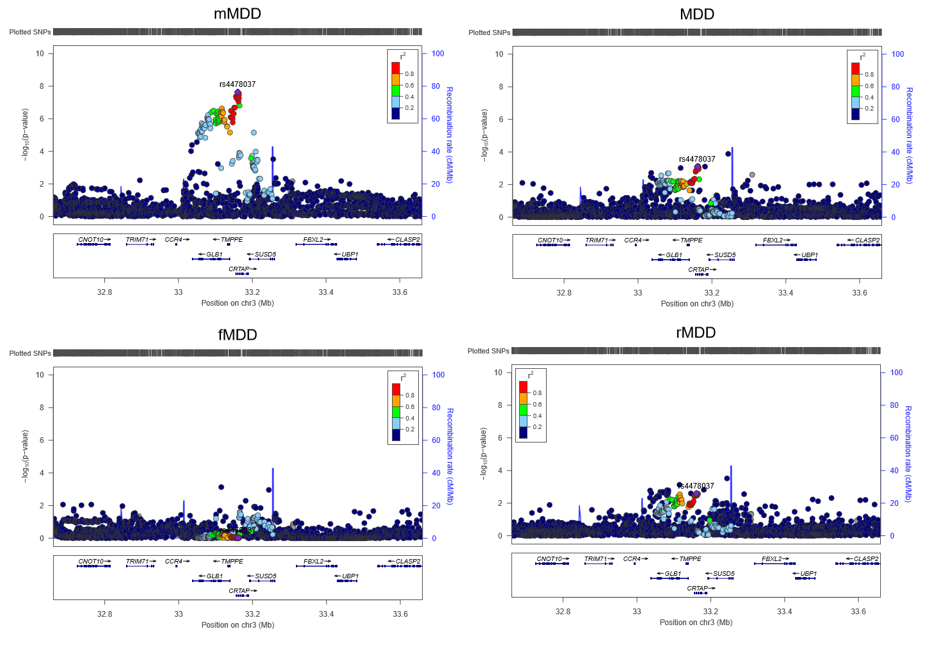


Supplementary Figure 4 - Regional association plot for rs4478037, an intronic SNP in *CRTAP*, and the top ranking SNP (P=2.37x10^-8^) in GWAS of depression in males only (mMDD), all cases and controls (MDD), recurrent cases and all controls (rMDD) and depression in females only (fMDD).


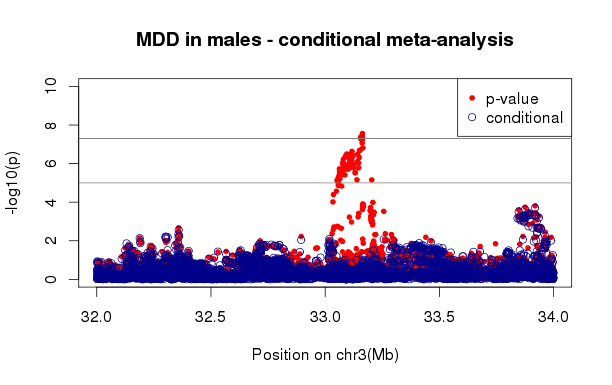


Supplementary Figure 5 - Manhattan plot of chromosome 3 from the GWAS of depression in males only. Red, filled points show meta-analysis p-values; blue, empty points show meta-analysis p-values conditional on SNPs with a multiple regression R2 > 0.9 with the top ranking SNP, rs4478037.

# Gene-based analysis

| **Gene** | **Chr** | **Start** | **Stop** | **NSNPs** | **NindSNPs** | **N** | **Stat** | **P-value** |
| --- | --- | --- | --- | --- | --- | --- | --- | --- |
| *CRTAP* | 3p22.3 | 33135450 | 33209265 | 190 | 76 | 19886 | 5.115 | 1.57x10-7 |
| *GLB1* | 3p22.3 | 33018100 | 33158694 | 450 | 157 | 19886 | 4.745 | 1.04x10-6 |
| *TMPPE* | 3p22.3 | 33111908 | 33158293 | 105 | 38 | 19886 | 4.701 | 1.29x10-6 |

Supplementary Table 12. Genome-wide significant (P<2.76x10-6) in the MAGMA gene-based analysis for depression in males only. NSNPS is the number of SNPs in the gene; NindSNPs is the number of independent SNPs in the gene.

# Pathway and functional genomic analysis

| **Original gene set ID** | **Original gene set description** | **Nominal P** | **FDR** | **Reconstituted gene set** |
| --- | --- | --- | --- | --- |
| GO:0031406 | carboxylic acid binding | 2.71x10^-8^ | <0.01 | *SLC5A, CRTAP, CASP5, SUSD5, GLB1, AQP3, GLO1, HTR1A, MS4A12, RAB26* |
| ENSG00000018236 | CNTN1 PPI subnetwork | 3.53x10^-7^ | <0.05 | *PLEKHB1, RAB6A, GLB1, MS4A12, CRTAP, PTPRG, PKD1, CHSY1, NDN, HTR1A* |

Supplementary Table 13. DEPICT reconstituted gene set enrichment results from independent suggestive SNPs (P<1x10^-5^) in males only meta-analyzed depression which survived multiple testing correction (FDR P<0.05).

| **Trait** | **SNP** | **cis-eQTL** | **Position Weight Matrix** | **Transcription Factor binding site** | **Histone Modifications** | **DNase hypersensitivity sites** | **FAIRE** | **DMR** |
| --- | --- | --- | --- | --- | --- | --- | --- | --- |
| MDD | rs2964802 | N | Y | N | N | N | N | N |
| MDD | rs56390503 | N | N | N | Y | Y | N | N |
| MDD | rs144735131 | Y | N | Y | Y | Y | N | N |
| MDD | rs140555 | Y | Y | N | Y | Y | N | N |
| MDD | rs10736455 | N | Y | Y | Y | Y | N | N |
| MDD | rs117715236 | N | Y | N | Y | N | N | N |
| MDD | rs4435501 | Y | N | Y | Y | Y | Y | N |
| MDD | rs12547341 | N | Y | N | N | N | N | N |
| MDD | rs80208956 | N | N | Y | Y | Y | N | N |
| MDD | rs73063271 | N | N | N | Y | Y | N | N |
| MDD | rs11743328 | Y | Y | N | N | N | N | N |
| MDD | rs115981586 | N | Y | N | N | N | N | N |
| MDD | rs145478122 | N | Y | N | Y | N | N | N |
| MDD | rs1874256 | N | Y | N | N | N | N | N |
| MDD | rs8050755 | N | Y | Y | Y | Y | N | N |
| MDD | rs73249855 | N | Y | Y | Y | Y | N | N |
| rMDD | rs4438172 | N | Y | N | N | N | N | N |
| rMDD | rs2291479 | N | N | Y | Y | Y | Y | N |
| rMDD | rs4941058 | N | Y | N | N | N | N | N |
| rMDD | rs13011737 | N | Y | N | N | N | N | N |
| rMDD | rs2964802 | N | Y | N | N | N | N | N |
| rMDD | rs8140097 | N | N | Y | N | N | N | N |
| rMDD | rs5760842 | Y | N | N | Y | Y | Y | N |
| rMDD | rs1792737 | N | Y | N | N | N | Y | N |
| rMDD | rs60716536 | N | Y | Y | N | Y | N | N |
| rMDD | rs2460546 | Y | Y | Y | N | N | N | N |
| rMDD | rs1031396 | N | Y | N | N | N | N | N |
| rMDD | rs79802045 | N | N | Y | Y | Y | N | N |
| rMDD | rs117901488 | Y | Y | N | N | N | N | N |
| rMDD | rs17615103 | N | N | Y | Y | N | N | N |
| rMDD | rs288342 | Y | N | N | Y | Y | N | N |
| rMDD | rs1486437 | N | Y | N | N | N | N | N |
| fMDD | rs17176546 | N | Y | N | Y | N | Y | N |
| fMDD | rs1448094 | Y | N | Y | Y | Y | N | N |
| fMDD | rs12464040 | Y | Y | N | N | N | Y | N |
| fMDD | rs79225303 | N | Y | N | Y | N | N | N |
| fMDD | rs61842316 | N | Y | N | N | Y | N | N |
| fMDD | rs111404313 | N | Y | N | Y | N | N | N |
| fMDD | rs1874256 | N | Y | N | N | N | N | N |
| fMDD | rs6893200 | N | Y | N | N | N | N | N |
| fMDD | rs73228161 | N | Y | N | Y | N | Y | N |
| fMDD | rs74031748 | N | Y | N | Y | N | N | N |
| fMDD | rs11613048 | N | Y | Y | Y | Y | Y | N |
| fMDD | rs79936639 | N | N | N | N | Y | N | N |
| fMDD | rs146951131 | N | N | N | N | Y | Y | N |
| mMDD | rs4478037 | Y | Y | N | N | N | N | N |
| mMDD | rs115736167 | N | Y | N | Y | Y | N | N |
| mMDD | rs113485090 | Y | Y | N | Y | N | N | N |
| mMDD | rs1380551 | N | Y | N | N | N | N | N |
| mMDD | rs117778141 | N | Y | N | Y | N | N | N |
| mMDD | rs10758202 | Y | N | Y | Y | Y | N | N |
| mMDD | rs150439352 | N | Y | N | N | N | N | N |
| mMDD | rs6822806 | N | Y | N | Y | Y | Y | N |
| mMDD | rs73108923 | N | N | N | N | N | N | N |
| mMDD | rs13127214 | N | N | N | Y | Y | N | N |
| mMDD | rs74002781 | Y | Y | Y | Y | Y | Y | N |
| mMDD | rs117621296 | N | Y | N | Y | Y | N | N |

Supplementary Table 14. Functional annotation of lead SNPs with meta-analysis P-value≤1x10-5. All information contained in this table was extracted from the GTEx database (<http://www.broadinstitute.org/gtex/>) and the Regulome DB database (<http://regulome.stanford.edu/index>). SNPs are ordered within MDD subtype by meta-analysis P-value. Only SNPs which returned a Regulome DB score of 1-6 are shown.

# Estimating SNP-based heritability using GREML

| **Cohort** | **Trait** | **h^2^_SNP_ (SE)** | **P-value** | **Unrelated N** |
| --- | --- | --- | --- | --- |
| GS:SFHS | MDD | 0.11 (0.12) | 0.16 | 7 795 |
|  | rMDD | 0.22 (0.15) | 0.07 | 7 562 |
|  | fMDD | 0.23 (0.14) | 0.05 | 5 662 |
|  | mMDD | 0 (0.19) | 0.5 | 4 740 |
| UKB | MDD | 0.20 (0.04) | 1.01x10-9 | 24 337 |
|  | rMDD | 0.20 (0.03) | 1.62x10-10 | 22 145 |
|  | fMDD | 0.22 (0.06) | 1.14x10-4 | 12 151 |
|  | mMDD | 0.18 (0.06) | 0.002 | 12 186 |

Supplementary Table 15. Output from GCTA-GREML using SNP data from all cases and controls (MDD), recurrent cases and all controls (rMDD), females only (fMDD) and males only (mMDD) in Generation Scotland (GS:SFHS) and UK Biobank (UKB). h^2^_SNP_ (SE) indicates the SNP-based heritability estimate on the liability scale and its standard error, P-value indicates the significance of the likelihood ratio test comparing the full and reduced models.

# Estimating polygenicity and SNP-based heritability using Linkage Disequilibrium Score Regression (LDSR)

| **Trait** | **h^2^_SNP_ (SE)** | **λ_GC_** | **μχ^2^** | **Intercept (SE)** | **Ratio (SE)** |
| --- | --- | --- | --- | --- | --- |
| GS:SFHS MDD | 0.12 (0.06) | 1.017 | 1.016 | 0.999 (0.007) | <0 |
| GS:SFHS rMDD | 0.16 (0.10) | 1.011 | 1.014 | 0.998 (0.007) | <0 |
| GS:SFHS fMDD | 0.20 (0.09) | 1.014 | 1.015 | 0.995 (0.007) | <0 |
| GS:SFHS mMDD | -0.07 (0.15) | 1.005 | 0.997 | 1.001 (0.006) | NA |
| UKB MDD | 0.11 (0.03) | 1.05 | 1.046 | 1.009 (0.006) | 0.19 (0.14) |
| UKB rMDD | 0.11 (0.02) | 1.056 | 1.048 | 1.007 (0.006) | 0.15 (0.13) |
| UKB fMDD | 0.11 (0.04) | 1.038 | 1.031 | 1.011 (0.006) | 0.34 (0.21) |
| UKB mMDD | 0.03 (0.04) | 1.023 | 1.019 | 1.013 (0.006) | 0.67 (0.32) |
| Meta-analyzed MDD | 0.12 (0.02) | 1.062 | 1.062 | 0.999 (0.006) | <0 |
| Meta-analyzed rMDD | 0.12 (0.02) | 1.062 | 1.059 | 1.001 (0.007) | 0.009 (0.11) |
| Meta-analyzed fMDD | 0.13 (0.03) | 1.035 | 1.038 | 0.998 (0.006) | <0 |
| Meta-analyzed mMDD | 0.05 (0.03) | 1.023 | 1.018 | 1.006 (0.006) | 0.35 (0.34) |

Supplementary Table 16. Output from univariate LDSR using summary statistics from GWAS of all cases and controls (MDD), recurrent cases and all controls (rMDD), females only (fMDD) and males only (mMDD) in Generation Scotland (GS:SFHS), UK Biobank (UKB) and meta-analysis of UKB and GS. h^2^_SNP_ (SE) indicates the SNP-based heritability estimate on the liability scale and its standard error; λ_GC_ indicates genomic control, estimated using median(chi^2)/0.4549; μχ^2^ indicates the mean chi-squared statistic; Intercept (SE) indicates the LDSR intercept with its standard error; and Ratio (SE) indicates proportion of inflation ascribed to causes other than polygenic heritability, estimated using (Intercept-1)/( μχ^2^-1), with its standard error.

# Genetic correlation with health related traits

| **Trait 1** | **Trait 2** | **PMID** | **rG** | **SE** | **Z** | **P-value** | **h^2^_obs_** | **h^2^_obs__se** | **h^2^_int_** | **h^2^_int__se** | **gcov_int** |
| --- | --- | --- | --- | --- | --- | --- | --- | --- | --- | --- | --- |
| **MDD** | **Neuroticism** | **27089181** | **0.67** | **0.07** | **9.37** | **7.06x10^-21^** | **0.09** | **0.008** | **0.99** | **0.012** | **0.102** |
| **MDD** | **Depressive symptoms** | **27089181** | **0.81** | **0.09** | **9.03** | **1.72x10^-19^** | **0.05** | **0.004** | **1.00** | **0.007** | **0.085** |
| **MDD** | **Subjective well being** | **27089181** | **-0.56** | **0.08** | **-7.14** | **9.12x10^-13^** | **0.03** | **0.002** | **1.00** | **0.008** | **-0.064** |
| **MDD** | **Age of first birth** | **27798627** | **-0.35** | **0.05** | **-6.37** | **1.92x10^-10^** | **0.06** | **0.004** | **0.95** | **0.008** | **-0.008** |
| **MDD** | **PGC major depressive disorder** | **22472876** | **0.67** | **0.12** | **5.47** | **4.57x10^-8^** | **0.17** | **0.026** | **1.01** | **0.006** | **0.007** |
| **MDD** | **PGC cross-disorder analysis** | **23453885** | **0.46** | **0.09** | **5.35** | **8.60x10^-8^** | **0.17** | **0.013** | **1.01** | **0.011** | **0.005** |
| **MDD** | **Bipolar disorder** | **21926972** | **0.32** | **0.08** | **4.09** | **4.35x10^-5^** | **0.43** | **0.038** | **1.02** | **0.008** | **0.005** |
| **MDD** | **Systemic lupus erythematosus** | **26502338** | **0.28** | **0.08** | **3.34** | **8.00x10^-4^** | **0.39** | **0.067** | **1.11** | **0.010** | **-0.006** |
| MDD | Number of children ever born | 27798627 | 0.21 | 0.07 | 3.01 | 0.003 | 0.03 | 0.002 | 0.97 | 0.007 | 0.000 |
| MDD | Chronotype | 27494321 | -0.19 | 0.07 | -2.78 | 0.006 | 0.10 | 0.006 | 1.01 | 0.008 | -0.011 |
| MDD | Years of schooling 2016 | 27225129 | -0.13 | 0.05 | -2.63 | 0.008 | 0.13 | 0.005 | 0.93 | 0.010 | -0.005 |
| MDD | Age at Menarche | 25231870 | -0.11 | 0.04 | -2.61 | 0.009 | 0.20 | 0.010 | 0.95 | 0.012 | 0.001 |
| MDD | Child birth length | 25281659 | -0.26 | 0.10 | -2.52 | 0.01 | 0.17 | 0.023 | 0.99 | 0.007 | 0.007 |
| MDD | College completion | 23722424 | -0.18 | 0.07 | -2.40 | 0.02 | 0.08 | 0.006 | 1.02 | 0.009 | 0.000 |
| MDD | Anorexia Nervosa | 24514567 | 0.16 | 0.07 | 2.33 | 0.02 | 0.55 | 0.032 | 0.90 | 0.007 | -0.003 |
| MDD | Ever vs never smoked | 20418890 | 0.19 | 0.09 | 2.26 | 0.02 | 0.07 | 0.007 | 1.00 | 0.006 | 0.006 |
| MDD | Extreme waist-to-hip ratio | 23563607 | 0.29 | 0.13 | 2.26 | 0.02 | 0.36 | 0.059 | 0.98 | 0.008 | -0.008 |
| MDD | Triglycerides in large VLDL | 27005778 | 0.25 | 0.11 | 2.26 | 0.02 | 0.12 | 0.029 | 0.99 | 0.006 | 0.003 |
| MDD | Concentration of chylomicrons  and largest VLDL particles | 27005778 | 0.30 | 0.14 | 2.16 | 0.03 | 0.12 | 0.027 | 0.99 | 0.007 | -0.002 |
| MDD | Total lipids in large VLDL | 27005778 | 0.24 | 0.11 | 2.13 | 0.03 | 0.14 | 0.030 | 0.98 | 0.006 | 0.002 |
| MDD | Ulcerative colitis | 26192919 | 0.16 | 0.08 | 2.07 | 0.04 | 0.25 | 0.032 | 1.05 | 0.010 | -0.006 |
| MDD | Triglycerides in medium VLDL | 27005778 | 0.26 | 0.12 | 2.07 | 0.04 | 0.10 | 0.030 | 1.00 | 0.006 | 0.002 |
| MDD | Phospholipids in large VLDL | 27005778 | 0.24 | 0.12 | 2.03 | 0.04 | 0.12 | 0.031 | 0.99 | 0.007 | 0.003 |
| MDD | Apolipoprotein A-I | 27005778 | 0.40 | 0.20 | 2.00 | 0.05 | 0.07 | 0.031 | 1.01 | 0.012 | -0.011 |
| MDD | Triglycerides in very large VLDL | 27005778 | 0.23 | 0.12 | 2.00 | 0.05 | 0.12 | 0.029 | 0.98 | 0.007 | 0.003 |
| MDD | Serum total triglycerides | 27005778 | 0.23 | 0.12 | 2.00 | 0.05 | 0.13 | 0.036 | 0.99 | 0.007 | 0.000 |
| MDD | Childhood obesity | 22484627 | 0.16 | 0.08 | 1.99 | 0.05 | 0.41 | 0.046 | 0.93 | 0.008 | 0.001 |
| MDD | Concentration of large VLDL particles | 27005778 | 0.24 | 0.12 | 1.99 | 0.05 | 0.14 | 0.035 | 0.97 | 0.007 | 0.001 |
| **rMDD** | **Neuroticism** | **27089181** | **0.65** | **0.07** | **9.50** | **2.01x10^-21^** | **0.09** | **0.006** | **0.99** | **0.011** | **0.122** |
| **rMDD** | **Depressive symptoms** | **27089181** | **0.79** | **0.09** | **8.60** | **7.65x10^-18^** | **0.05** | **0.004** | **1.00** | **0.008** | **0.105** |
| **rMDD** | **Subjective well being** | **27089181** | **-0.54** | **0.08** | **-6.68** | **2.43x10^-11^** | **0.03** | **0.002** | **1.00** | **0.008** | **-0.076** |
| **rMDD** | **PGC major depressive disorder** | **22472876** | **0.67** | **0.15** | **4.58** | **4.59x10^-6^** | **0.17** | **0.031** | **1.01** | **0.008** | **0.010** |
| **rMDD** | **PGC cross-disorder analysis** | **23453885** | **0.44** | **0.10** | **4.38** | **1.19x10^-5^** | **0.17** | **0.014** | **1.02** | **0.012** | **0.014** |
| **rMDD** | **Bipolar disorder** | **21926972** | **0.30** | **0.08** | **3.63** | **3.00x10^-4^** | **0.46** | **0.040** | **1.02** | **0.008** | **0.009** |
| **rMDD** | **Age of first birth** | **27798627** | **-0.23** | **0.07** | **-3.40** | **7.00x10^-4^** | **0.06** | **0.004** | **0.95** | **0.009** | **-0.014** |
| rMDD | Child birth length | 25281659 | -0.31 | 0.11 | -2.83 | 0.005 | 0.18 | 0.024 | 0.99 | 0.007 | 0.007 |
| rMDD | Number of children ever born | 27798627 | 0.19 | 0.07 | 2.69 | 0.007 | 0.02 | 0.002 | 0.98 | 0.007 | 0.000 |
| rMDD | College completion | 23722424 | -0.21 | 0.08 | -2.66 | 0.008 | 0.08 | 0.006 | 1.02 | 0.009 | 0.007 |
| rMDD | Concentration of chylomicrons  and largest VLDL particles | 27005778 | 0.39 | 0.15 | 2.59 | 0.01 | 0.12 | 0.031 | 0.99 | 0.007 | -0.006 |
| rMDD | Ulcerative colitis | 26192919 | 0.21 | 0.08 | 2.53 | 0.01 | 0.26 | 0.033 | 1.05 | 0.011 | -0.006 |
| rMDD | Ever vs never smoked | 20418890 | 0.24 | 0.10 | 2.39 | 0.02 | 0.07 | 0.007 | 1.00 | 0.007 | 0.001 |
| rMDD | Inflammatory Bowel Disease (Euro) | 26192919 | 0.17 | 0.07 | 2.37 | 0.02 | 0.34 | 0.037 | 1.06 | 0.011 | -0.006 |
| rMDD | Total lipids in medium VLDL | 27005778 | 0.29 | 0.12 | 2.35 | 0.02 | 0.16 | 0.038 | 0.98 | 0.008 | -0.003 |
| rMDD | Triglycerides in medium VLDL | 27005778 | 0.29 | 0.12 | 2.31 | 0.02 | 0.11 | 0.035 | 1.00 | 0.008 | 0.000 |
| rMDD | Serum total triglycerides | 27005778 | 0.28 | 0.12 | 2.28 | 0.02 | 0.14 | 0.039 | 0.99 | 0.009 | -0.004 |
| rMDD | Triglycerides in large VLDL | 27005778 | 0.25 | 0.11 | 2.21 | 0.03 | 0.13 | 0.034 | 0.99 | 0.008 | 0.002 |
| rMDD | Total lipids in large VLDL | 27005778 | 0.26 | 0.12 | 2.21 | 0.03 | 0.15 | 0.035 | 0.98 | 0.008 | 0.001 |
| rMDD | Chronotype | 27494321 | -0.16 | 0.07 | -2.18 | 0.03 | 0.10 | 0.006 | 1.02 | 0.009 | -0.015 |
| rMDD | Years of schooling 2016 | 27225129 | -0.11 | 0.05 | -2.18 | 0.03 | 0.13 | 0.005 | 0.93 | 0.010 | -0.003 |
| rMDD | Phospholipids in medium VLDL | 27005778 | 0.27 | 0.12 | 2.15 | 0.03 | 0.13 | 0.037 | 0.99 | 0.008 | -0.002 |
| rMDD | Age at Menarche | 25231870 | -0.12 | 0.05 | -2.14 | 0.03 | 0.20 | 0.011 | 0.95 | 0.012 | -0.002 |
| rMDD | Total lipids in small HDL | 27005778 | 0.49 | 0.24 | 2.09 | 0.04 | 0.05 | 0.028 | 1.00 | 0.008 | -0.009 |
| rMDD | Anorexia Nervosa | 24514567 | 0.14 | 0.07 | 2.06 | 0.04 | 0.55 | 0.032 | 0.91 | 0.008 | -0.001 |
| rMDD | Free cholesterol in medium VLDL | 27005778 | 0.26 | 0.13 | 2.03 | 0.04 | 0.13 | 0.037 | 0.99 | 0.008 | -0.002 |
| rMDD | Concentration of medium VLDL particles | 27005778 | 0.23 | 0.12 | 2.03 | 0.04 | 0.16 | 0.040 | 0.98 | 0.008 | 0.000 |
| rMDD | Concentration of small VLDL particles | 27005778 | 0.25 | 0.12 | 2.01 | 0.04 | 0.17 | 0.040 | 0.99 | 0.009 | -0.005 |
| rMDD | Triglycerides in small VLDL | 27005778 | 0.25 | 0.12 | 2.00 | 0.05 | 0.13 | 0.038 | 0.99 | 0.009 | -0.003 |
| rMDD | Triglycerides in very small VLDL | 27005778 | 0.27 | 0.14 | 1.98 | 0.05 | 0.16 | 0.040 | 1.00 | 0.010 | -0.006 |
| rMDD | Triglycerides in small HDL | 27005778 | 0.34 | 0.17 | 1.98 | 0.05 | 0.07 | 0.028 | 1.00 | 0.009 | -0.004 |
| **fMDD** | **Neuroticism** | **27089181** | **0.65** | **0.10** | **6.57** | **5.17x10^-11^** | **0.09** | **0.008** | **0.99** | **0.012** | **0.068** |
| **fMDD** | **Depressive symptoms** | **27089181** | **0.76** | **0.12** | **6.18** | **6.58x10^-10^** | **0.05** | **0.004** | **1.00** | **0.007** | **0.062** |
| **fMDD** | **Subjective well being** | **27089181** | **-0.62** | **0.11** | **-5.66** | **1.49x10^-8^** | **0.03** | **0.002** | **1.00** | **0.008** | **-0.036** |
| **fMDD** | **PGC cross-disorder analysis** | **23453885** | **0.39** | **0.10** | **3.90** | **9.81x10^-5^** | **0.17** | **0.013** | **1.01** | **0.011** | **0.003** |
| **fMDD** | **Bipolar disorder** | **21926972** | **0.31** | **0.09** | **3.54** | **4.00x10^-4^** | **0.43** | **0.038** | **1.02** | **0.008** | **0.001** |
| **fMDD** | **PGC major depressive disorder** | **22472876** | **0.52** | **0.15** | **3.54** | **4.00x10^-4^** | **0.17** | **0.026** | **1.01** | **0.006** | **0.009** |
| fMDD | Age of first birth | 27798627 | -0.23 | 0.07 | -3.15 | 0.002 | 0.06 | 0.004 | 0.95 | 0.008 | -0.020 |
| fMDD | Ever vs never smoked | 20418890 | 0.29 | 0.10 | 2.95 | 0.003 | 0.07 | 0.007 | 1.00 | 0.006 | 0.000 |
| fMDD | Systemic lupus erythematosus | 26502338 | 0.25 | 0.10 | 2.62 | 0.009 | 0.39 | 0.067 | 1.11 | 0.010 | -0.001 |
| fMDD | Years of schooling 2016 | 27225129 | -0.15 | 0.06 | -2.55 | 0.01 | 0.13 | 0.005 | 0.93 | 0.010 | -0.007 |
| fMDD | Chronotype | 27494321 | -0.18 | 0.08 | -2.22 | 0.03 | 0.10 | 0.006 | 1.01 | 0.008 | -0.006 |
| fMDD | College completion | 23722424 | -0.19 | 0.09 | -2.15 | 0.03 | 0.08 | 0.006 | 1.02 | 0.009 | -0.003 |
| fMDD | Acetoacetate | 27005778 | 0.42 | 0.20 | 2.04 | 0.04 | 0.07 | 0.028 | 0.98 | 0.007 | -0.011 |
| fMDD | Number of children ever born | 27798627 | 0.18 | 0.09 | 2.01 | 0.04 | 0.03 | 0.002 | 0.97 | 0.007 | 0.000 |
| mMDD | Neuroticism | 27089181 | 1.05 | 0.43 | 2.46 | 0.01 | 0.09 | 0.008 | 0.99 | 0.012 | 0.079 |
| mMDD | Depressive symptoms | 27089181 | 1.29 | 0.53 | 2.45 | 0.01 | 0.05 | 0.004 | 1.00 | 0.007 | 0.060 |
| mMDD | PGC major depressive disorder | 22472876 | 1.38 | 0.61 | 2.27 | 0.02 | 0.17 | 0.026 | 1.01 | 0.006 | -0.001 |
| mMDD | Age of first birth | 27798627 | -0.77 | 0.34 | -2.26 | 0.02 | 0.06 | 0.004 | 0.95 | 0.008 | 0.009 |
| mMDD | Subjective well being | 27089181 | -0.67 | 0.34 | -1.97 | 0.05 | 0.03 | 0.002 | 1.00 | 0.007 | -0.058 |

Supplementary Table 17. Results which showed nominal significance (P<0.05) from bivariate Linkage Disequilibrium Score Regression (LDSR) between MDD subtypes (Trait 1, all cases and controls (MDD), recurrent cases and all controls (rMDD), females only (fMDD) and males only (mMDD)) and 200 health-related traits (Trait 2), ordered by P-value. Traits which survive false-discovery rate multiple testing correction for 800 tests are highlighted in bold. PMID refers to the PubMed Identification number from which the GWAS summary statistics were derived; rG denotes the estimated genetic correlation between traits; SE denotes the standard error of the rG estimate; Z denotes Z-score; P-value denotes association P-value for the rG estimate; h^2^_obs_ denotes the observed heritability of the health-related trait, as calculated by LDSR, h^2^_obs__se denotes the standard error of the h^2^_obs_ estimate; h^2^_int_ denotes the single-trait LDSR incepted for the health-related trait; h^2^_int__se denotes the standard error for the h^2^_int_ estimate; gcov_int denotes the cross-trait LDSR intercept.

# Polygenic profiling analysis

P-values presented below have been adjusted using FDR correction, accounting for 80 tests.

## MDD in GS:SFHS using PGC weights for major depressive disorder

| **Trait** | **PGS P_T_** | **OR(CI)** | **Z-ratio** | **F statistic  (df,dendf)** | **P-value** | **% variance explained** |
| --- | --- | --- | --- | --- | --- | --- |
| MDD | **1** | **1.18 (1.14,1.23)** | **7.83** | **61.3 (1,14229.1)** | **5.92x10^-14^** | **0.37** |
|  | **0.5** | **1.21 (1.17,1.26)** | **9.15** | **83.7 (1,14289.9)** | **5.34x10^-18^** | **0.51** |
|  | **0.1** | **1.20 (1.16,1.25)** | **8.62** | **74.4 (1,14432.5)** | **2.89x10^-16^** | **0.46** |
|  | **0.05** | **1.19 (1.14,1.23)** | **7.88** | **62.1 (1,14399.3)** | **4.75x10^-14^** | **0.39** |
|  | **0.01** | **1.16 (1.12,1.21)** | **6.70** | **44.8 (1,14575.4)** | **1.37x10^-10^** | **0.31** |
| rMDD | **1** | **1.23 (1.17,1.30)** | **7.40** | **54.8 (1,13323.3)** | **1.27x10^-12^** | **0.36** |
|  | **0.5** | **1.26 (1.20,1.32)** | **8.22** | **67.6 (1,13355.0)** | **5.76x10^-15^** | **0.45** |
|  | **0.1** | **1.25 (1.19,1.32)** | **7.93** | **62.8 (1,13528.7)** | **3.94x10^-14^** | **0.42** |
|  | **0.05** | **1.23 (1.16,1.29)** | **7.11** | **50.6 (1,13474.6)** | **8.65x10^-12^** | **0.34** |
|  | **0.01** | **1.19 (1.12,1.26)** | **5.66** | **32.1 (1,13686.9)** | **8.11x10^-8^** | **0.24** |
| fMDD | **1** | **1.19(1.14,1.25)** | **6.85** | **46.9 (1,9500.8)** | **5.27x10^-11^** | **0.49** |
|  | **0.5** | **1.23(1.17,1.28)** | **7.95** | **63.3 (1,9492.1)** | **3.94x10^-14^** | **0.66** |
|  | **0.1** | **1.22(1.16,1.28)** | **7.75** | **60.0 (1,9404.2)** | **1.05x10^-13^** | **0.63** |
|  | **0.05** | **1.21(1.15,1.27)** | **7.29** | **53.2 (1,9454.3)** | **2.63x10^-12^** | **0.57** |
|  | **0.01** | **1.19(1.13,1.25)** | **6.39** | **40.8 (1,9557.5)** | **1.01x10^-9^** | **0.47** |
| mMDD | **1** | **1.15(1.07,1.23)** | **3.68** | **13.6 (1,6696.6)** | **0.001** | **0.19** |
|  | **0.5** | **1.18(1.10,1.26)** | **4.46** | **19.9 (1,6701.5)** | **4.24x10^-5^** | **0.28** |
|  | **0.1** | **1.15(1.07,1.23)** | **3.59** | **12.9 (1,6780.3)** | **0.001** | **0.18** |
|  | **0.05** | **1.13(1.04,1.21)** | **3.02** | **9.12 (1,6712.9)** | **0.01** | **0.13** |
|  | 0.01 | 1.11(1.02,1.19) | 2.43 | 5.90 (1,6669.0) | 0.06 | 0.09 |

Supplementary Table 18. Results from polygenic profiling analysis of MDD subtypes (all cases and controls (MDD), recurrent cases and all controls (rMDD), females only (fMDD) and males only (mMDD)) in GS:SFHS using weights from the PGC GWAS of major depressive disorder.

## MDD in GS:SFHS using UKB weights for major depressive disorder

| **Trait** | **PGS P_T_** | **OR(CI)** | **Z-ratio** | **F statistic  (df,dendf)** | **P-value** | **% variance explained** |
| --- | --- | --- | --- | --- | --- | --- |
| MDD | **1** | **1.10 (1.05,1.14)** | **4.37** | **19.1 (1,14496.6)** | **4.27x10^-4^** | **0.11** |
|  | **0.5** | **1.10 (1.05,1.14)** | **4.32** | **18.6 (1,14490.3)** | **4.27x10^-4^** | **0.11** |
|  | **0.1** | **1.08 (1.03,1.12)** | **3.52** | **12.4 (1,14666.2)** | **0.004** | **0.07** |
|  | **0.05** | **1.08 (1.04,1.13)** | **3.77** | **14.2 (1,14839.9)** | **0.003** | **0.08** |
|  | **0.01** | **1.08 (1.04,1.13)** | **3.71** | **13.8 (1,14585.9)** | **0.003** | **0.08** |
| rMDD | **1** | **1.11 (1.05,1.17)** | **3.62** | **13.1 (1,13611.8)** | **0.003** | **0.08** |
|  | **0.5** | **1.11 (1.05,1.17)** | **3.56** | **12.7 (1,13613.3)** | **0.004** | **0.08** |
|  | **0.1** | **1.08 (1.02,1.14)** | **2.76** | **7.63 (1,13770.2)** | **0.03** | **0.05** |
|  | **0.05** | **1.10 (1.03,1.15)** | **2.93** | **8.61 (1,13859.7)** | **0.02** | **0.05** |
|  | **0.01** | **1.09 (1.03,1.15)** | **3.00** | **8.98 (1,13605.8)** | **0.02** | **0.06** |
| fMDD | 1 | 1.06 (1.01,1.12) | 2.38 | 5.66 (1,9482.0) | 0.08 | 0.06 |
|  | 0.5 | 1.06 (1.01,1.11) | 2.19 | 4.78 (1,9456.5) | 0.12 | 0.05 |
|  | 0.1 | 1.06 (1.01,1.11) | 2.23 | 4.98 (1,9589.7) | 0.11 | 0.05 |
|  | 0.05 | 1.06 (1.01,1.12) | 2.38 | 5.66 (1,9706.4) | 0.08 | 0.06 |
|  | 0.01 | 1.07 (1.01,1.12) | 2.45 | 5.98 (1,9618.5) | 0.07 | 0.06 |
| mMDD | **1** | **1.17 (1.09,1.25)** | **4.19** | **17.5 (1,6792.2)** | **5.74x10^-4^** | **0.23** |
|  | **0.5** | **1.17 (1.10,1.25)** | **4.40** | **19.4 (1,6797.2)** | **4.27x10^-4^** | **0.25** |
|  | **0.1** | **1.11 (1.04,1.19)** | **2.92** | **8.51 (1,6789.7)** | **0.02** | **0.11** |
|  | **0.05** | **1.12 (1.04,1.20)** | **3.05** | **9.33 (1,6801.6)** | **0.02** | **0.12** |
|  | **0.01** | **1.11 (1.04,1.19)** | **2.89** | **8.34 (1,6707.4)** | **0.02** | **0.11** |

Supplementary Table 19. Results from polygenic profiling analysis of MDD subtypes in GS:SFHS using weights from the UKB GWAS of major depressive disorder.

## MDD in UKB using PGC weights for major depressive disorder

| **Trait** | **PGS P_T_** | **OR(CI)** | **T value** | **P-value** | **%**  **variance**  **explained** |
| --- | --- | --- | --- | --- | --- |
| MDD | **1** | **1.64 (1.40,1.92)** | **5.89** | **1.92x10^-8^** | **0.51** |
|  | **0.5** | **1.24 (1.19,1.29)** | **11.07** | **2.85x10^-27^** | **0.58** |
|  | **0.1** | **1.25 (1.21,1.30)** | **11.86** | **1.78x10^-30^** | **0.53** |
|  | **0.05** | **1.21 (1.17,1.25)** | **11.34** | **2.75x10^-28^** | **0.27** |
|  | **0.01** | **1.16 (1.09,1.16)** | **8.06** | **5.84x10^-15^** | **0.08** |
| rMDD | **1** | **1.68 (1.42,1.97)** | **5.72** | **4.99x10^-8^** | **0.57** |
|  | **0.5** | **1.27 (1.22,1.32)** | **11.18** | **9.79x10^-28^** | **0.63** |
|  | **0.1** | **1.28 (1.23,1.33)** | **11.79** | **2.21x10^-30^** | **0.57** |
|  | **0.05** | **1.23 (1.19,1.28)** | **11.23** | **6.72x10^-28^** | **0.32** |
|  | **0.01** | **1.15 (1.11,1.18)** | **8.46** | **2.52x10^-16^** | **0.08** |
| fMDD | **1** | **1.82 (1.44,2.32)** | **5.05** | **2.00x10^-6^** | **0.49** |
|  | **0.5** | **1.23 (1.17,1.29)** | **7.71** | **9.40x10^-14^** | **0.72** |
|  | **0.1** | **1.28 (1.21,1.34)** | **9.32** | **1.58x10^-19^** | **0.66** |
|  | **0.05** | **1.23 (1.18,1.29)** | **8.92** | **5.28x10^-18^** | **0.36** |
|  | **0.01** | **1.14 (1.10,1.19)** | **6.55** | **3.10x10^-10^** | **0.15** |
| mMDD | **1** | **1.52 (1.19,1.89)** | **3.29** | **0.004** | **0.56** |
|  | **0.5** | **1.27 (1.20,1.34)** | **8.23** | **1.59x10^-15^** | **0.48** |
|  | **0.1** | **1.25 (1.18,1.32)** | **7.62** | **1.66x10^-13^** | **0.43** |
|  | **0.05** | **1.20 (1.15,1.26)** | **7.20** | **3.67x10^-12^** | **0.21** |
|  | **0.01** | **1.12 (1.07,1.17)** | **5.00** | **2.43x10^-6^** | **0.02** |

Supplementary Table 20. Results from polygenic profiling analysis of MDD subtypes in UKB using weights from the PGC GWAS of major depressive disorder.

## MDD in UKB using GS:SFHS weights for major depressive disorder

| **Trait** | **PGS P_T_** | **OR(CI)** | **T value** | **P-value** | **%**  **variance**  **explained** |
| --- | --- | --- | --- | --- | --- |
| MDD | **1** | **1.06 (1.03,1.09)** | **4.28** | **4.45x10^-4^** | **0.07** |
|  | **0.5** | **1.06 (1.03,1.09)** | **4.19** | **4.45x10^-4^** | **0.06** |
|  | **0.1** | **1.05 (1.02,1.08)** | **3.71** | **0.002** | **0.06** |
|  | **0.05** | **1.05 (1.03,1.08)** | **3.85** | **0.001** | **0.00** |
|  | 0.01 | 1.02 (0.99,1.04) | 1.10 | 1.00 | 0.08 |
| rMDD | **1** | **1.06 (1.03,1.09)** | **4.09** | **5.46x10^-4^** | **0.07** |
|  | **0.5** | **1.06 (1.03,1.09)** | **4.07** | **5.46x10^-4^** | **0.04** |
|  | **0.1** | **1.05 (1.02,1.08)** | **3.00** | **0.02** | **0.04** |
|  | **0.05** | **1.04 (1.01,1.07)** | **2.90** | **0.02** | **0.00** |
|  | 0.01 | 0.99 (0.97,1.03) | -0.46 | 1.00 | 0.08 |
| fMDD | **1** | **1.08 (1.04,1.12)** | **4.22** | **4.45x10^-4^** | **0.15** |
|  | **0.5** | **1.08 (1.04,1.12)** | **4.22** | **4.45x10^-4^** | **0.12** |
|  | **0.1** | **1.07 (1.03,1.11)** | **3.80** | **0.001** | **0.15** |
|  | **0.05** | **1.08 (1.04,1.12)** | **4.23** | **4.45x10^-4^** | **0.01** |
|  | 0.01 | 1.02 (0.98,1.06) | 1.11 | 1.00 | 0.15 |
| mMDD | 1 | 1.03 (0.99,1.07) | 1.47 | 0.87 | 0.01 |
|  | 0.5 | 1.03 (0.99,1.07) | 1.27 | 1.00 | 0.01 |
|  | 0.1 | 1.02 (0.98,1.06) | 0.39 | 1.00 | 0.00 |
|  | 0.05 | 1.01 (0.97,1.05) | 0.62 | 1.00 | 0.00 |
|  | 0.01 | 1.00 (0.96,1.04) | 0.08 | 1.00 | 0.02 |

Supplementary Table 21. Results from polygenic profiling analysis of MDD subtypes in UKB using weights from the GS:SFHS GWAS of major depressive disorder.

# Major Depressive Disorder Working Group of the Psychiatric Genomics Consortium Authorship

Stephan Ripke 1,2,3, Manuel Mattheisen 4,5,11, Abdel Abdellaoui 7, Mark J Adams 8, Esben Agerbo 9,10,11, Tracy M Air 12, Till F M Andlauer 13,14, Silviu-Alin Bacanu 15, Marie Bækvad-Hansen 11,16, Aartjan T F Beekman 17, David A Bennett 18, Klaus Berger 19, Tim B Bigdeli 15,20, Jonas Bybjerg-Grauholm 11,16, Enda M Byrne 21, Na Cai 22, Enrique Castelao 23, Toni-Kim Clarke 8, Jonathan R I Coleman 24, CONVERGE Consortium 25, Baptiste Couvy-Duchesne 26,27, Nick Craddock 28, Udo Dannlowski 30, Gareth Davies 31, Gail Davies 32, EJC de Geus 7,33, Philip De Jager 34, Ian J Deary 32, Franziska Degenhardt 35,36, Nese Direk 37,38, Erin C Dunn 39,40,41, Erik A Ehli 31, Thalia C Eley 24, Valentina Escott-Price 42, Tõnu Esko 1,43,44,45, Hilary K Finucane 46,47, Andreas J Forstner 35,36,48,49, Josef Frank 50, Michael Gill 51, Scott D Gordon 52, Jakob Grove 4,5,11,53, Lynsey S Hall 8,54, Thomas F Hansen 55,56, Christine Søholm Hansen 11,16, Thomas F Hansen 57, Caroline Hayward 58, Andrew C Heath 59, Anjali K Henders 21, Stefan Herms 35,36,49, Ian B Hickie 60, Per Hoffmann 35,36,49, Albert Hofman 38, Georg Homuth 61, Carsten Horn 62, Jouke-Jan Hottenga 7, David Hougaard 11,16, Hailiang Huang 2,39,63, Marcus Ising 64, Rick Jansen 17, Eric Jorgenson 65, Stefan Kloiber 64,66,67, James A Knowles 68, Warren W. Kretzschmar 69, Jesper Krogh 70, Zoltán Kutalik 71,72, Glyn Lewis 73, Yihan Li 69, Donald J MacIntyre 74,75, Pamela AF Madden 59, Jonathan Marchine 76, Hamdi Mbarek 7, Patrick McGrath 79, Peter McGuffin 24, Sarah Elizabeth Medland 80, Divya Mehta 81, Andres Metspalu 45,82, Christel M Middeldorp 7, Evelin Mihailov 45,83, Yuri Milaneschi 17, Lili Milani 45, Grant W Montgomery 84, Sara Mostafavi 85,86, Niamh Mullins 24, Matthias Nauck 87,88, Bernard Ng 86, Merete Nordentoft 11,89, Dale R Nyholt 90, Michael C O'Donovan 91, Paul F O'Reilly 24, Hogni Oskarsson 92, Michael J Owen 91, Sara A Paciga 93, Carsten Bøcker Pedersen 9,11,94, Marianne Giørtz Pedersen 9,11,94, Nancy L Pedersen 95, Michele L Pergadia 96, Roseann E. Peterson 15,97, Erik Pettersson 98, Wouter J Peyrot 17, Giorgio Pistis 23, David J Porteous 99, Danielle Posthuma 100,101, James B Potash 102, Jorge A Quiroz 103, John P Rice 59, Brien P. Riley 15, Margarita Rivera 24,104, Douglas M. Ruderfer 105, Saira Saeed Mirza 38, Robert Schoevers 106, Thomas G Schulze 50,107,108,109,110, Ling Shen 65, Jianxin Shi 111, Engilbert Sigurdsson 112, Grant C B Sinnamon 113, Johannes H Smit 17, Daniel J Smith 114, Hreinn Stefansson 115, Stacy Steinberg 115, Fabian Streit 50, Jana Strohmaier 50, Katherine E Tansey 116, Alexander Teumer 117, Wesley Thompson 56,99,118,119,120, Pippa A Thomson 99, Thorgeir E Thorgeirsson 121, Jens Treutlein 50, Maciej Trzaskowski 122, André G Uitterlinden 123, Daniel Umbricht 124, Sandra Van der Auwera 125, Gerard van Grootheest 17, Albert M van Hemert 126, Alexander Viktorin 95, Henry Völzke 117, Yunpeng Wang 11,56,119, Bradley T. Webb 127, Myrna M Weissman 79,128, Jürgen Wellmann 19, Gonneke Willemsen 7, Stephanie H Witt 50, Hualin S Xi 129, Bernhard T Baune 12, Douglas H R Blackwood 8, Dorret I Boomsma 7, Henriette N Buttenschøn 5,11,130, Sven Cichon 35,131,132,133, Enrico Domenici 134, Jonathan Flint 69,135, Hans J Grabe 125, Steven P Hamilton 136, Kenneth S Kendler 15, Qingqin S Li 137, Susanne Lucae 64, Patrik K Magnusson 95, Nicholas G Martin 52,138, Andrew M McIntosh 8,32, Ole Mors 11,139, Preben Bo Mortensen 5,10,11,140, Bertram Müller-Myhsok 13,14,141, Markus M Nöthen 35,36, Brenda WJH Penninx 17, Roy H Perlis 40,142, Martin Preisig 23, Marcella Rietschel 50, Catherine Schaefer 65, Jordan W Smoller 39,40,41, Kari Stefansson 115, Henning Tiemeier 38,143,144, Rudolf Uher 145, Thomas Werge 56,118,146, Ashley R Winslow 147,148, Gerome Breen 24,149, Douglas F Levinson 150, Cathryn M Lewis 24,151, Naomi R Wray 21,122, Anders D Børglum 4,5,11, Patrick F Sullivan 98,152,153

1, Medical and Population Genetics, Broad Institute, Cambridge, US

2, Analytic and Translational Genetics Unit, Massachusetts General Hospital, Boston, US

3, Department of Psychiatry and Psychotherapy, Universitätsmedizin Berlin Campus Charité Mitte, Berlin, DE

4, Department of Biomedicine, Aarhus University, Aarhus, DK

5, iSEQ, Centre for Integrative Sequencing, Aarhus University, Aarhus, DK

6, iSPYCH, The Lundbeck Foundation Initiative for Integrative Psychiatric Research, Aarhus, DK

7, Dept of Biological Psychology & EMGO+ Institute for Health and Care Research, Vrije Universiteit Amsterdam, Amsterdam, NL

8, Division of Psychiatry, University of Edinburgh, Edinburgh, GB

9, Centre for Integrated Register-based Research, Aarhus University, Aarhus, DK

10, National Centre for Register-Based Research, Aarhus University, Aarhus,

11, iPSYCH, The Lundbeck Foundation Initiative for Integrative Psychiatric Research, Aarhus, DK

12, Discipline of Psychiatry, University of Adelaide, Adelaide, AU

13, Department of Translational Research in Psychiatry, Max Planck Institute of Psychiatry, Munich, DE

14, Munich Cluster for Systems Neurology (SyNergy), Munich, DE

15, Department of Psychiatry, Virginia Commonwealth University, Richmond, US

16, Center for Neonatal Screening, Department for Congenital Disorders, Statens Serum Institut, Copenhagen, DK

17, Department of Psychiatry, Vrije Universiteit Medical Center and GGZ inGeest, Amsterdam, NL

18, Rush Alzheimer's Disease Center, Rush University Medical Center, Chicago, US

19, Institute of Epidemiology and Social Medicine, University of Muenster, Muenster, DE

20, Virginia Institute for Psychiatric and Behavior Genetics, Richmond, US

21, Queensland Brain Institute, The University of Queensland, Brisbane, AU

22, Human Genetics, Wellcome Trust Sanger Institute, Cambridge, GB

23, Department of Psychiatry, University Hospital of Lausanne, Prilly, CH

24, MRC Social Genetic and Developmental Psychiatry Centre, King's College London, London, GB

25, University of Oxford,

26, Centre for Advanced Imaging, The University of Queensland, Saint Lucia, AU

27, Queensland Brain Institute, The University of Queensland, Saint Lucia, AU

28, Psychological Medicine, Cardiff University, Cardiff, GB

30, Department of Psychiatry, University of Münster, Münster, DE

31, Avera Institute for Human Genetics, Sioux Falls, US

32, Centre for Cognitive Ageing and Cognitive Epidemiology, University of Edinburgh, Edinburgh, GB

33, Amsterdam Public Health institute, Vrije Universiteit Medical Center, Amsterdam, NL

34, Neurology, Brigham and Women's Hospital, Boston, US

35, Institute of Human Genetics, University of Bonn, Bonn, DE

36, Life&Brain Center, Department of Genomics, University of Bonn, Bonn, DE

37, Psychiatry, Dokuz Eylul University School Of Medicine, Izmir, TR

38, Epidemiology, Erasmus MC, Rotterdam, NL

39, Stanley Center for Psychiatric Research, Broad Institute, Cambridge, US

40, Department of Psychiatry, Massachusetts General Hospital, Boston, US

41, Psychiatric and Neurodevelopmental Genetics Unit (PNGU), Massachusetts General Hospital, Boston, US

42, Neuroscience and Mental Health, Cardiff University, Cardiff, GB

43, Division of Endocrinology, Children's Hospital Boston, Boston, US

44, Department of Genetics, Harvard Medical School, Boston, US

45, Estonian Genome Center, University of Tartu, Tartu, EE

46, Department of Epidemiology, Harvard T.H. Chan School of Public Health, Boston, US

47, Department of Mathematics, Massachusetts Institute of Technology, Cambridge, US

48, Department of Psychiatry (UPK), University of Basel, Basel, CH

49, Human Genomics Research Group, Department of Biomedicine, University of Basel, Basel, CH

50, Department of Genetic Epidemiology in Psychiatry, Central Institute of Mental Health, Mannheim, DE

51, Department of Psychiatry, Trinity College Dublin, Dublin, IE

52, Genetics and Computational Biology, QIMR Berghofer Medical Research Institute, Brisbane, AU

53, Bioinformatics Research Centre (BiRC), Aarhus University, Aarhus, DK

54, Institute of Genetic Medicine, Newcastle University, Newcastle upon Tyne, GB

55, Headache Centre, Department of Neurology, Rigshospitalet, Glostrup, DK

56, Institute of Biological Psychiatry, Mental Health Center Sct. Hans, Mental Health Services Capital Region of Denmark, Copenhagen, DK

57, iPSYCH, The Lundbeck Foundation Initiative for Psychiatric Research, Copenhagen, DK

58, Medical Research Council Human Genetics Unit, Institute of Genetics and Molecular Medicine, University of Edinburgh, Edinburgh, GB

59, Department of Psychiatry, Washington University in Saint Louis School of Medicine, Saint Louis, US

60, Brain and Mind Centre, University of Sydney, Sydney, AU

61, Interfaculty Institute for Genetics and Functional Genomics, Department of Functional Genomics, University Medicine and Ernst Moritz Arndt University Greifswald, Greifswald, DE

62, Roche Pharmaceutical Research and Early Development, Pharmaceutical Sciences, Roche Innovation Center Basel, F. Hoffmann-La Roche Ltd, Basel, CH

63, Department of Medicine, Harvard Medical School, Boston, US

64, Max Planck Institute of Psychiatry, Munich, DE

65, Division of Research, Kaiser Permanente Northern California, Oakland, US

66, Centre for Addiction and Mental Health, Toronto, CA

67, Department of Psychiatry, University of Toronto, Toronto, CA

68, Psychiatry & The Behavioral Sciences, University of Southern California, Los Angeles, US

69, Wellcome Trust Centre for Human Genetics, University of Oxford, Oxford, GB

70, Department of Endocrinology at Herlev University Hospital, University of Copenhagen, Copenhagen, DK

71, Swiss Institute of Bioinformatics, Lausanne, CH

72, Institute of Social and Preventive Medicine (IUMSP), University Hospital of Lausanne, Lausanne, CH

73, Division of Psychiatry, University College London, London, GB

74, Mental Health, NHS 24, Glasgow, GB

75, Division of Psychiatry, Centre for Clinical Brain Sciences, University of Edinburgh, Edinburgh, GB

76, Statistics, University of Oxford, Oxford, GB

79, Psychiatry, Columbia University College of Physicians and Surgeons, New York, US

80, Genetics and Computational Biology, QIMR Berghofer Medical Research Institute, Herston, AU

81, School of Psychology and Counseling, Queensland University of Technology, Brisbane, AU

82, Institute of Molecular and Cell Biology, University of Tartu, Tartu, EE

83, Estonian Biocentre, Tartu, EE

84, Institute for Molecular Biology, University of Queensland, Brisbane, AU

85, Medical Genetics, University of British Columbia, Vancouver, CA

86, Statistics, University of British Columbia, Vancouver, CA

87, DZHK (German Centre for Cardiovascular Research), Partner Site Greifswald, University Medicine, Matthias Nauck, Greifswald, DE

88, Institute of Clinical Chemistry and Laboratory Medicine, University Medicine Greifswald, Greifswald, DE

89, Mental Health Centre Copenhagen, Copenhagen Universtity Hospital, Copenhagen, DK

90, Institute of Health and Biomedical Innovation, Queensland University of Technology, Brisbane, AU

91, MRC Centre for Neuropsychiatric Genetics and Genomics, Cardiff University, Cardiff, GB

92, Humus, Reykjavik, IS

93, Human Genetics and Computational Biomedicine, Pfizer Global Research and Development, Groton, US

94, National Centre for Register-Based Research, Aarhus University,

95, Department of Medical Epidemiology and Biostatistics, Karolinska Institutet, Stockholm, SE

96, Charles E. Schmidt College of Medicine, Florida Atlantic University, Boca Raton, US

97, Virginia Institute for Psychiatric & Behavioral Genetics, Virginia Commonwealth University, Richmond, US

98, Medical Epidemiology and Biostatistics, Karolinska Institutet, Stockholm, SE

99, Medical Genetics Section, CGEM, IGMM, University of Edinburgh, Edinburgh, GB

100, Complex Trait Genetics, Vrije Universiteit Amsterdam, Amsterdam, NL

101, Clinical Genetics, Vrije Universiteit Medical Center, Amsterdam, NL

102, Psychiatry, University of Iowa, Iowa City, US

103, Solid GT, Boston, US

104, Department of Biochemistry and Molecular Biology II, Institute of Neurosciences, Center for Biomedical Research, University of Granada, Granada, ES

105, Psychiatry, Icahn School of Medicine at Mount Sinai, New York, US

106, Department of Psychiatry, University of Groningen, University Medical Center Groningen, Groningen, NL

107, Department of Psychiatry and Behavioral Sciences, Johns Hopkins University, Baltimore, US

108, Institute of Psychiatric Phenomics and Genomics (IPPG), Medical Center of the University of Munich, Campus Innenstadt, Munich, DE

109, Human Genetics Branch, NIMH Division of Intramural Research Programs, Bethesda, US

110, Department of Psychiatry and Psychotherapy, University Medical Center Göttingen, Goettingen, DE

111, Division of Cancer Epidemiology and Genetics, National Cancer Institute, Bethesda, US

112, Faculty of Medicine, Department of Psychiatry, School of Health Sciences, University of Iceland, Reykjavik, IS

113, School of Medicine and Dentistry, James Cook University, Townsville, AU

114, Institute of Health and Wellbeing, University of Glasgow, Glasgow, GB

115, deCODE Genetics / Amgen, Reykjavik, IS

116, College of Biomedical and Life Sciences, Cardiff University, Cardiff, GB

117, Institute for Community Medicine, University Medicine Greifswald, Greifswald, DE

118, iPSYCH, The Lundbeck Foundation Initiative for Integrative Psychiatric Research, Copenhagen, DK

119, KG Jebsen Centre for Psychosis Research, Norway Division of Mental Health and Addiction, Oslo University Hospital, Oslo, NO

120, Department of Psychiatry, University of California, San Diego, San Diego, US

121, deCODE genetics / Amgen, Reykjavik, IS

122, Institute for Molecular Bioscience, The University of Queensland, Brisbane, AU

123, Internal Medicine, Erasmus MC, Rotterdam, NL

124, Roche Pharmaceutical Research and Early Development, Neuroscience, Ophthalmology and Rare Diseases Discovery & Translational Medicine Area, Roche Innovation Center Basel , F. Hoffmann-La Roche Ltd, Basel, CH

125, Department of Psychiatry and Psychotherapy, University Medicine Greifswald, Greifswald, DE

126, Department of Psychiatry, Leiden University Medical Center, Leiden, NL

127, Virginia Institute of Psychiatric & Behavioral Genetics , Virginia Commonwealth University, Richmond, US

128, Division of Epidemiology, New York State Psychiatric Institute, New York, US

129, Computational Sciences Center of Emphasis, Pfizer Global Research and Development, Cambridge, US

130, Department of Clinical Medicine, Translational Neuropsychiatry Unit, Aarhus University, Aarhus, DK

131, Institute of Neuroscience and Medicine (INM-1), Research Center Juelich, Juelich, DE

132, Department of Biomedicine, University of Basel, Basel, CH

133, Division of Medical Genetics, University of Basel, Basel, CH

134, Centre for Integrative Biology , Università degli Studi di Trento, Trento, IT

135, Psychiatry, University of California Los Angeles, Los Angeles, US

136, Psychiatry, Kaiser Permanente Northern California, San Francisco, US

137, Neuroscience Therapeutic Area, Janssen Research and Development, LLC, Titusville, US

138, School of Psychology, The University of Queensland, Brisbane, AU

139, Psychosis Research Unit, Aarhus University Hospital, Risskov, Aarhus, DK

140, National Centre for Register-based Research, Aarhus University, Aarhus, DK

141, University of Liverpool, Liverpool, GB

142, Psychiatry, Harvard Medical School, Boston, US

143, Child and Adolescent Psychiatry, Erasmus MC, Rotterdam, NL

144, Psychiatry, Erasmus MC, Rotterdam, NL

145, Psychiatry, Dalhousie University, Halifax, CA

146, Institute of Clinical Medicine , University of Copenhagen, Copenhagen, DK

147, Human Genetics and Computational Biomedicine, Pfizer Global Research and Development, Cambridge, US

148, Orphan Disease Center, Perelman School of Medicine, University of Pennsylvania, Philadelphia, US

149, NIHR BRC for Mental Health, King's College London, London, GB

150, Psychiatry & Behavioral Sciences, Stanford University, Stanford, US

151, Department of Medical & Molecular Genetics , King's College London, London, GB

152, Genetics, University of North Carolina at Chapel Hill, Chapel Hill, US

153, Psychiatry, University of North Carolina at Chapel Hill, Chapel Hill, US

Version: 3rd February, 2016

# References

1. Smith BH, Campbell A, Linksted P, Fitzpatrick B, Jackson C, Kerr SM, et al. Cohort Profile: Generation Scotland: Scottish Family Health Study (GS:SFHS). The study, its participants and their potential for genetic research on health and illness. Int J Epidemiol. 2013;42(3):689-700.

2. Smith BH, Campbell H, Blackwood D, Connell J, Connor M, Deary IJ, et al. Generation Scotland: the Scottish Family Health Study; a new resource for researching genes and heritability. BMC Med Genet. 2006;7:74.

3. Kerr SM, Campbell A, Murphy L, Hayward C, Jackson C, Wain LV, et al. Pedigree and genotyping quality analyses of over 10,000 DNA samples from the Generation Scotland: Scottish Family Health Study. BMC Med Genet. 2013;14:38.

4. Gunderson KL. Whole-genome genotyping on bead arrays. Methods Mol Biol. 2009;529:197-213.

5. First MB, Spitzer RL, Gibbon M, Williams JB. Structured Clinical Interview for DSM-IV Axis I Disorders, Clinician Version (SCID-CV). Washington, DC, USA: American Psychiatric Publishing, Inc. 1997.

6. Fernandez-Pujals AM, Adams MJ, Thomson P, McKechanie AG, Blackwood DH, Smith BH, et al. Epidemiology and Heritability of Major Depressive Disorder, Stratified by Age of Onset, Sex, and Illness Course in Generation Scotland: Scottish Family Health Study (GS:SFHS). PLoS One. 2015;10(11):e0142197.

7. Sudlow C, Gallacher J, Allen N, Beral V, Burton P, Danesh J, et al. UK Biobank: An Open Access Resource for Identifying the Causes of a Wide Range of Complex Diseases of Middle and Old Age. Plos Med. 2015;12(3).

8. UK Biobank. UK Biobank: Protocol for a large-scale prospective epidemiological resource 2007 [Available from: <http://www.ukbiobank.ac.uk/wp-content/uploads/2011/11/UK-Biobank-Protocol.pdf?phpMyAdmin=trmKQlYdjjnQIgJ%2CfAzikMhEnx6>.

9. UK Biobank. Touchscreen questionnaire 2012 [Available from: <http://www.ukbiobank.ac.uk/wp-content/uploads/2011/06/Touch_screen_questionnaire.pdf?phpMyAdmin=trmKQlYdjjnQIgJ%2CfAzikMhEnx6>.

10. Spitzer RL, Kroenke K, Williams JBW, Primary PHQ. Validation and utility of a self-report version of PRIME-MD - The PHQ primary care study. Jama-J Am Med Assoc. 1999;282(18):1737-44.

11. Smith DJ, Nicholl BI, Cullen B, Martin D, Ul-Haq Z, Evans J, et al. Prevalence and Characteristics of Probable Major Depression and Bipolar Disorder within UK Biobank: Cross-Sectional Study of 172,751 Participants. Plos One. 2013;8(11).

12. Kessler RC, Berglund P, Demler O, Jin R, Koretz D, Merikangas KR, et al. The epidemiology of major depressive disorder - Results from the National Comorbidity Survey Replication (NCS-R). Jama-J Am Med Assoc. 2003;289(23):3095-105.

13. Altamura AC, Carta MG, Carpiniello B, Piras A, Maccio MV, Marcia L. Lifetime prevalence of brief recurrent depression (results from a community survey). Eur Neuropsychopharmacol. 1995;5 Suppl:99-102.

14. Weissman MM, Bland RC, Canino GJ, Faravelli C, Greenwald S, Hwu HG, et al. Cross-national epidemiology of major depression and bipolar disorder. JAMA. 1996;276(4):293-9.

15. Office for National Statistics. Overview of the UK population - Office for National Statistics [Available from: [www.ons.gov.uk/peoplepopulationandcommunity/populationandmigration/populationestimates/articles/overviewoftheukpopulation/february2016](http://www.ons.gov.uk/peoplepopulationandcommunity/populationandmigration/populationestimates/articles/overviewoftheukpopulation/february2016).

16. Yang J, Zaitlen NA, Goddard ME, Visscher PM, Price AL. Advantages and pitfalls in the application of mixed-model association methods. Nat Genet. 2014;46(2):100-6.

17. Yang J, Lee SH, Goddard ME, Visscher PM. GCTA: a tool for genome-wide complex trait analysis. Am J Hum Genet. 2011;88(1):76-82.

18. Zaitlen N, Kraft P, Patterson N, Pasaniuc B, Bhatia G, Pollack S, et al. Using Extended Genealogy to Estimate Components of Heritability for 23 Quantitative and Dichotomous Traits. Plos Genet. 2013;9(5).

19. Lynch M, Walsh B. Genetics and analysis of quantitative traits. Sunderland, Mass.: Sinauer; 1998. xvi, 980 pages p.

20. Visscher PM, Hemani G, Vinkhuyzen AAE, Chen GB, Lee SH, Wray NR, et al. Statistical Power to Detect Genetic (Co)Variance of Complex Traits Using SNP Data in Unrelated Samples. Plos Genet. 2014;10(4).

21. Cortes A, Hadler J, Pointon JP, Robinson PC, Karaderi T, Leo P, et al. Identification of multiple risk variants for ankylosing spondylitis through high-density genotyping of immune-related loci. Nature Genetics. 2013;45(7):730-+.

22. Pers TH, Karjalainen JM, Chan Y, Westra HJ, Wood AR, Yang J, et al. Biological interpretation of genome-wide association studies using predicted gene functions. Nat Commun. 2015;6.

23. Chang CC, Chow CC, Tellier LCAM, Vattikuti S, Purcell SM, Lee JJ. Second-generation PLINK: rising to the challenge of larger and richer datasets. Gigascience. 2015;4.

24. 1000 Genomes Project Consortium, Abecasis GR, Auton A, Brooks LD, DePristo MA, Durbin RM, et al. An integrated map of genetic variation from 1,092 human genomes. Nature. 2012;491(7422):56-65.

25. Barrett T, Wilhite SE, Ledoux P, Evangelista C, Kim IF, Tomashevsky M, et al. NCBI GEO: archive for functional genomics data sets--update. Nucleic Acids Res. 2013;41(Database issue):D991-5.

26. Flicek P, Amode MR, Barrell D, Beal K, Billis K, Brent S, et al. Ensembl 2014. Nucleic Acids Res. 2014;42(Database issue):D749-55.

27. Ashburner M, Ball CA, Blake JA, Botstein D, Butler H, Cherry JM, et al. Gene ontology: tool for the unification of biology. The Gene Ontology Consortium. Nat Genet. 2000;25(1):25-9.

28. Kanehisa M, Goto S, Sato Y, Furumichi M, Tanabe M. KEGG for integration and interpretation of large-scale molecular data sets. Nucleic Acids Res. 2012;40(Database issue):D109-14.

29. Croft D, O'Kelly G, Wu G, Haw R, Gillespie M, Matthews L, et al. Reactome: a database of reactions, pathways and biological processes. Nucleic Acids Res. 2011;39(Database issue):D691-7.

30. Lage K, Karlberg EO, Storling ZM, Olason PI, Pedersen AG, Rigina O, et al. A human phenome-interactome network of protein complexes implicated in genetic disorders. Nat Biotechnol. 2007;25(3):309-16.

31. Blake JA, Bult CJ, Eppig JT, Kadin JA, Richardson JE, Mouse Genome Database G. The Mouse Genome Database: integration of and access to knowledge about the laboratory mouse. Nucleic Acids Res. 2014;42(Database issue):D810-7.

32. Boyle AP, Hong EL, Hariharan M, Cheng Y, Schaub MA, Kasowski M, et al. Annotation of functional variation in personal genomes using RegulomeDB. Genome Res. 2012;22(9):1790-7.

33. Visscher PM. A note on the asymptotic distribution of likelihood ratio tests to test variance components. Twin Res Hum Genet. 2006;9(4):490-5.
